# Environmental Limitations and Interspecific Interactions Across the Distribution Range of Gerbils

**DOI:** 10.1002/ece3.72468

**Published:** 2025-11-10

**Authors:** Jiuqi Zhao, Rong Zhang, Yiqiang Dong, Mengyue Wang, Wenshan Chen, Die Chen, Suwen Yang

**Affiliations:** ^1^ College of Grassland Xinjiang Agricultural University Urumqi China; ^2^ College of Grassland Inner Mongolia Agricultural University Hohhot Inner Mongolia China; ^3^ Key Laboratory of Grassland Resources and Ecology Urumqi Xinjiang China; ^4^ Key Laboratory of Grassland Resources and Ecology Ministry of Education Urumqi China

**Keywords:** environmental adaptability, gerbil, interspecific interactions, joint species distribution model, maxent model

## Abstract

To elucidate the environmental adaptability and interspecific relationships of gerbil species, we analyzed the distribution data of seven species using the Maximum Entropy model (MaxEnt) and Joint Species Distribution Models (JSDMs) in relation to environmental variables. The MaxEnt results indicated high model accuracy, with training area under the receiver operating characteristic curve (AUC) values ranging from 0.939 to 0.998 and testing AUC values ranging from 0.896 to 0.994. Except for Mongolian Gerbil ( *Meriones unguiculatus* ) AUC indicating “good” performance, all species reached an “excellent” level of accuracy; model accuracy evaluation showed that the training TSS values ranged from 0.339 to 0.841 and the testing TSS values from 0.319 to 0.960, indicating good predictive performance. Jackknife tests revealed substantial interspecific differences in responses to environmental variables. Precipitation‐related factors (bio12, bio13, bio14, bio18, bio19) and temperature‐related factors (bio4, bio5, bio6, bio7, bio9) were the primary drivers of species distributions, while topographic variables (slope, aspect, elevation) and anthropogenic factors also played important roles. The JSDMs analysis further highlighted species‐specific responses to environmental factors and uncovered strong residual correlations among species. Notably, Mid‐day Gerbil ( *Meriones meridianus* ) and Libyan Jird ( *Meriones libycus* ) (−0.91), as well as Mongolian Gerbil and Great Gerbil ( *Rhombomys opimus* ) (−0.82), exhibited significant exclusionary relationships. In contrast, Tamarisk Gerbil ( *Meriones tamariscinus* ) and Przewalski's Jird ( *Brachiones przewalskii* ) (0.72), as well as Tamarisk Gerbil and Mid‐day Gerbil (0.66), showed evidence of positive associations. In summary, this study not only clarifies the environmental adaptability of gerbil rodents in the arid regions of Central Asia but also reveals their potential interspecific interaction mechanisms. These findings provide new insights into the dynamics of desert rodent communities and offer valuable implications for the management of rodent pests in arid ecosystems.

## Graphical abstract

This study explores the environmental adaptability and interspecific relationships among seven gerbil species across arid regions of Central Asia. By integrating Maxent and Joint Species Distribution Models, we reveal species‐specific habitat preferences and key ecological factors shaping community assembly and coexistence in desert ecosystems.

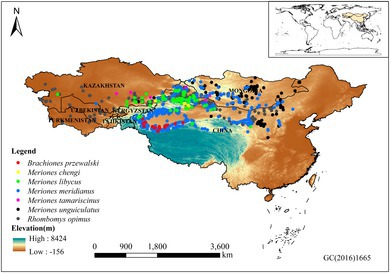

## 1 Introduction

Gerbils are typical desert and semi‐desert rodents, widely distributed across the arid regions of Central Asia and the Mongolian–Xinjiang arid zone (Zhou et al. 2001). They play important roles in maintaining ecosystem functions, such as seed dispersal (Li et al. 2025) and soil disturbance (Su 2016). Due to the fragility of arid ecosystems, gerbil populations are highly sensitive to climate change and environmental variation. Their distribution patterns are influenced not only by abiotic factors, including temperature, precipitation, and soil type (Chi et al. 2024), but also by biotic interactions, such as interspecific competition, predation, and coexistence mechanisms (Li et al. 2024). Therefore, investigating the environmental adaptability and interspecific interactions of gerbils is essential for understanding the formation of rodent communities and their ecological roles in arid ecosystems.

This study focuses on seven dominant gerbil species widely distributed across Central Asia and the Mongolian–Xinjiang arid regions, including Mid‐day Gerbil, Tamarisk Gerbil, Mongolian Gerbil, Libyan Jird, Cheng's Gerbil, Great Gerbil, and Przewalski's Jird. Although these species share similar ecological adaptations to arid environments, they exhibit distinct ecological habits. Mid‐day Gerbil typically inhabits fixed or semi‐fixed sand dunes in desert regions, is active both day and night, and primarily feeds on plant seeds. Tamarisk Gerbil prefers densely vegetated areas, is nocturnal, and mainly granivorous. Libyan Jird and Cheng's Gerbil are commonly found in desert steppe habitats. Great Gerbil mainly occupies Haloxylon‐dominated desert areas, is diurnal, and exhibits omnivorous feeding behavior; Przewalski's Jird is largely confined to sandy deserts (Wang 1983), while Mongolian Gerbil occurs mainly in semi‐desert grasslands, is diurnal, and also omnivorous (Liu et al. 2017). Despite their shared ecological traits, these species display notable differences in spatial distribution and environmental preferences, which may facilitate coexistence through niche differentiation or positive interspecific interactions. However, most previous studies have concentrated on the responses of single species to environmental factors (Qiao et al. 2021; Wang, Wang, et al. 2022; Wang, Zhang, and Zhao 2022), with few investigations examining, at the community level, the distribution patterns of multiple species under the combined influence of abiotic and biotic drivers and their potential interspecific interactions.

In recent years, species distribution models (SDMs) have been widely used to examine the relationships between species distributions and environmental factors. Models based on the maximum entropy principle (MaxEnt) perform well even with limited sample sizes (Hu et al. 2020) and have been applied in studies of desert rodents (Gan et al. 2022; Qiao et al. 2021; Wang, Wang, et al. 2022; Wang, Zhang, and Zhao 2022). However, traditional SDMs often overlook interspecific interactions, which may lead to underestimation of dynamic changes in ecological niches (Wang, Wang, et al. 2022; Wang, Zhang, and Zhao 2022). Joint Species Distribution Models (JSDMs) address this limitation by simultaneously considering the spatial distributions of multiple species, thereby revealing competitive relationships and facilitative interactions (Warton et al. 2015), and offering a novel approach to understanding community structure and coexistence mechanisms.

Building on this, the present study integrates geographic distribution data for seven gerbil species with multiple environmental variables, employing a combined SDMs and JSDMs approach to: (1) identify the key abiotic factors influencing species distributions; (2) quantify potential facilitative or competitive interactions among species; and (3) investigate the mechanisms underlying multi‐species coexistence in arid regions. The findings are expected to provide a theoretical basis for rodent management and biodiversity conservation in arid ecosystems, as well as a reference for predicting the potential impacts of climate change on biodiversity.

## 2 Materials and Methods

### 2.1 Study Areas and Species Occurrence Sites

**FIGURE 1:**
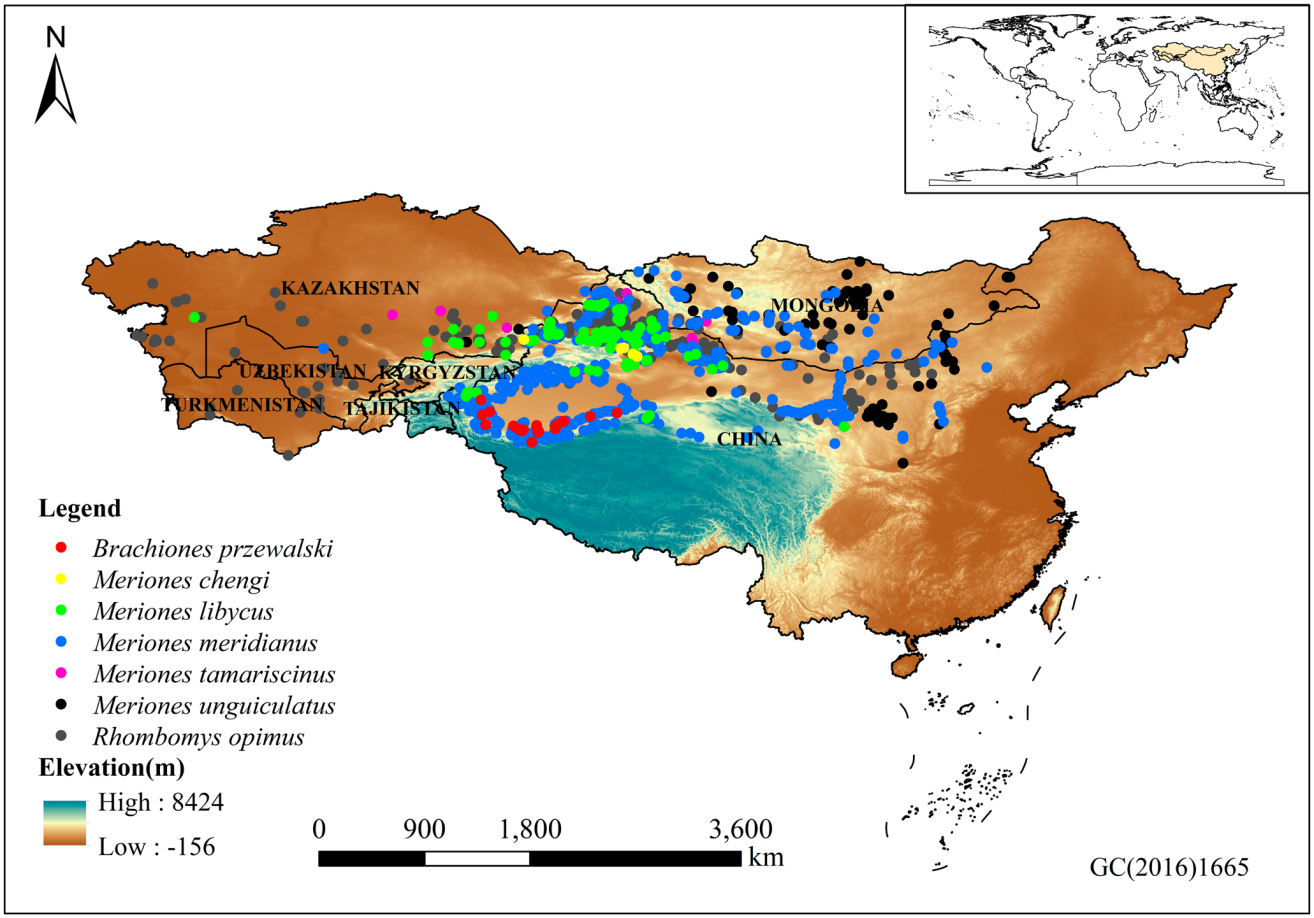
Species occurrence points used in MaxEnt modeling.

The study area was defined as the desert regions of China, Mongolia, Kazakhstan, Turkmenistan, Kyrgyzstan, Uzbekistan, and Tajikistan (Figure 1), as these regions represent the primary distribution range of the seven gerbil species under investigation (Smith et al. 2010; Wilson 1993).

**FIGURE 2:**
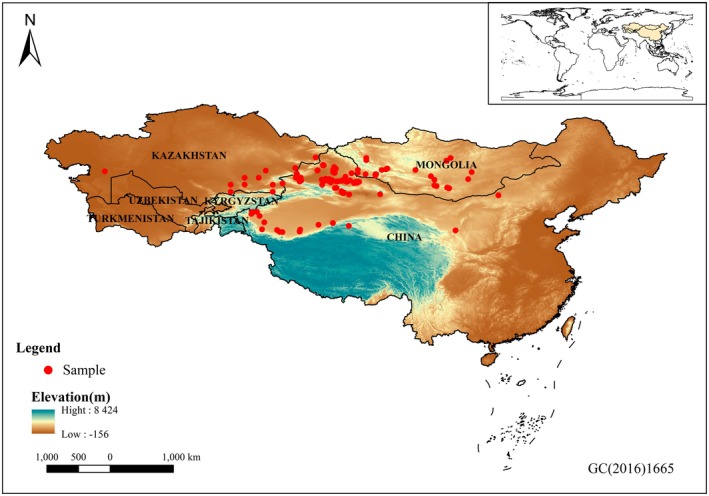
Species occurrence points used in JSDM modeling.

Acquisition of gerbil distribution data: (1) From 2022 to 2023, field surveys were conducted across various prefectures in Xinjiang, China, to obtain geographic distribution records of seven gerbil species occurring in the region. (2) Additional occurrence data were compiled from the Global Biodiversity Information Facility (GBIF; www.gbif.org) and published literature (Qiao et al. 2021; Wang, Wang, et al. 2022; Wang, Zhang, and Zhao 2022; Zheng et al. 2015; Ma 2023) from both domestic and international sources. For records that provided only locality names without geographic coordinates, coordinates were retrieved using the GPSSPG website (http://www.gpsspg.com/). These data covered the distribution of the seven gerbil species in other provinces of China outside Xinjiang, as well as in Mongolia, Kazakhstan, Turkmenistan, Kyrgyzstan, Uzbekistan, and Tajikistan. To reduce spatial autocorrelation in the species distribution data, we used the ENMTools.pl. software to automatically identify the spatial resolution and filter redundant records within the same grid cell. After processing, a total of 756 occurrence points were retained for Mid‐day Gerbil ( *Meriones meridianus* ), 804 for Great Gerbil ( *Rhombomys opimus* ), 127 for Libyan Jird ( *Meriones libycus* ), 27 for Tamarisk Gerbil ( *Meriones tamariscinus* ), 22 for Przewalski's Jird ( *Brachiones przewalskii* ), 114 for Mongolian Gerbil ( *Meriones unguiculatus* ), and 10 for Cheng's Gerbil( *Meriones chengi* ). These geographic occurrence records were used for MaxEnt modeling (Figure 1 and Table S1). In addition, when two or more species were recorded at the same location, their occurrence records were merged and converted into presence/absence (PA) data for use in the joint species distribution models (JSDMs), resulting in a total of 120 geographic sites (Figure 2 and Table S2).

### 2.2 Acquisition and Analysis of Environmental Variables

A total of 25 environmental variables related to climate, topography, and human disturbance were initially selected as predictors for modeling. The climatic variables consisted of 19 bioclimatic factors obtained from the WorldClim data set. The topographic variables included elevation, slope, and aspect. Human disturbance variables comprised grazing intensity (https://noda.ac.cn/aircas), the nighttime light index (Li et al. 2020), and the human footprint index (Mu et al. 2022). Both the bioclimatic and elevation data were sourced from WorldClim (https://www.worldclim.org), while slope and aspect were derived from elevation data using the “3D Analyst” toolbox in ArcGIS 10.8 (Koutrotsios et al. 2020).

The 19 bioclimatic variables obtained from the WorldClim database encompass annual means, extremes, and seasonal variability of temperature and precipitation, thereby providing a comprehensive representation of the climatic conditions that species rely on for survival (Hijmans et al. 2005). Climate is one of the most influential environmental factors shaping species distributions. This is particularly true in arid desert regions, where precipitation and temperature directly determine vegetation growth and water availability, and consequently exert indirect effects on food resources and habitat conditions for rodents (Elith and Leathwick 2009).

Topographic factors indirectly influence species distributions by altering temperature, humidity, solar radiation, and vegetation patterns (Körner 2007). Among them, elevation determines climatic gradients and reflects the spatial variation of temperature and precipitation with altitude. Slope affects soil moisture retention and vegetation cover, thereby modifying habitat suitability. Aspect determines solar radiation and hydrothermal conditions, which play a critical role in vegetation growth and the distribution of rodent habitats in arid regions (Singh 2020).

Anthropogenic disturbance factors, such as grazing intensity, nighttime light index, and human footprint index, play important roles in shaping rodent habitats. Grazing intensity reflects the level of grassland resource utilization, directly affecting vegetation biomass and community structure, which in turn influences food availability and burrow habitats for rodents (Su 2016). The night‐time light index serves as an indirect indicator of human economic activity and urbanization, reflecting the degree of habitat fragmentation and disturbance caused by human activities (Huang et al. 2023). The human footprint index integrates the cumulative pressures of roads, settlements, and land use on natural environments, providing a key measure of human disturbance intensity. In summary, climatic factors represent the macro‐environmental context of species distributions, topographic factors capture local microenvironmental variation, and anthropogenic disturbance factors reveal the impacts of human activities on habitat suitability (Table S3).

### 2.3 Maxent Model Optimization and Implementation

A total of 25 environmental variables were selected for the modeling analysis, including 19 bioclimatic variables, 3 topographic variables, and 3 anthropogenic variables. All variables were resampled to a spatial resolution of 1 km using ArcGIS 10.8 to ensure consistency across data sets (Zhang, Ci, et al. 2023). The layers were then masked and clipped based on the world administrative boundary vector data and converted into ASCII format compatible with the MaxEnt model. To minimize the potential effects of spatial autocorrelation among species occurrence points, a spatial thinning process was applied using a grid cell size of 1 km × 1 km, ensuring that only one occurrence record was retained within each grid cell. The raster and terra packages in R were used to unify the spatial resolution and projection of all environmental variables and to construct an environmental variable stack for modeling. Model performance was evaluated using multiple metrics, including the area under the receiver operating characteristic curve (AUC) (Webb and Ting 2005), the true skill statistic (TSS) (Farashi and Alizadeh‐Noughani 2023) and the corrected Akaike Information Criterion (AICc) (Shi et al. 2023; Zhao et al. 2021) and variable importance was assessed using permutation tests. To reduce the effects of multicollinearity, highly correlated variables were excluded based on Spearman correlation coefficients (Liu et al. 2020; Chi et al. 2024). Subsequently, model parameters were optimized using the ENMeval package, testing six feature combinations (L, LQ, H, LQH, LQHP, LQHPT) and regularization multipliers (RM) ranging from 0.5 to 10, incremented by 0.5. Ten‐fold cross‐validation, ΔAICc, and AUC metrics were used to select the optimal model (Shi et al. 2023; Zhao et al. 2021). Species occurrence data and environmental variables were imported into MaxEnt 3.4.4 (Phillips et al. 2006) for modeling (Gan et al. 2024), with occurrence points randomly split into 75% training and 25% testing data sets, a widely used ratio that balances model calibration and independent evaluation (Phillips et al. 2006). To ensure adequate environmental sampling, 10,000 background points were set, and each model was run 10 times with cross‐validation to ensure stability (Naeem et al. 2024).

During modeling, Jackknife analysis was performed to assess the relative contribution of each environmental variable to species distributions (Zhang, Chen, et al. 2023). Finally, model predictions were evaluated using the receiver operating characteristic (ROC) curve (Xian et al. 2022), with AUC values ranging from 0 to 1, where higher values indicate better predictive performance. According to common classification standards, AUC values are interpreted as poor (0–0.6), fair (0.6–0.7), moderate (0.7–0.8), good (0.8–0.9), and excellent (0.9–1) (Wu, Goldfeld, et al. 2024; Wu, Li, et al. 2024; Zhang et al. 2022).

### 2.4 Joint Species Distribution Modeling

#### 2.4.1 Model Construction Method

In this study, Joint Species Distribution Models (JSDMs) were used to analyze the relationships between species and environmental variables. Based on a Bayesian framework, a multi‐species binomial regression model was constructed using the jSDM_binomial_probit function in the jSDM R package. This model employs a Probit link function, suitable for handling presence/absence (PA) data, and simultaneously estimates species–environment responses and residual covariances among species within the Bayesian hierarchical structure (Chib and Greenberg 1998; Van Ee et al. 2022). The response variable consisted of presence/absence data for each species at each survey site, and environmental variables with contributions greater than 5% were considered the primary factors influencing species distributions (Lu et al. 2025). Environmental variables were included as fixed effects to quantify their influence on species distributions, while site effects were treated as random effects to capture variability among locations. Latitude and longitude were incorporated as latent variables to account for spatial autocorrelation (Cranston et al. 2024). Model parameters were estimated using Bayesian inference via Markov Chain Monte Carlo (MCMC) methods, allowing posterior distributions to be obtained and the statistical associations between species distributions and environmental factors to be evaluated (Zhu et al. 2018).

#### 2.4.2 Model Fit Performance Evaluation

In this study, the Markov Chain Monte Carlo (MCMC) method was used for Bayesian inference to ensure the stability of the model and the convergence of parameter estimates. To optimize model fit, the burn‐in period and sampling iterations were carefully set to minimize the influence of initial values on parameter estimates and to improve the reliability of the posterior distributions. Initial values for model parameters, including fixed effects (*β*), random effects (*α*), and latent variables (*λ*), were set to ensure the stability of MCMC sampling. Using Bayesian inference, species effects and the influence of environmental variables were sampled, and parameter estimates were iteratively optimized to enhance model convergence. Convergence of the MCMC sampling was evaluated using visualization tools such as trace plots and density plots (Robert et al. 2018).

### 2.5 Joint Species Distribution Model Analysis

This study quantifies the response of each species to different environmental variables by estimating the beta coefficients. The sign of the beta coefficient reflects the direction of the environmental factor's influence on species distribution. A positive beta value indicates that the environmental variable facilitates species distribution, while a negative beta value suggests that the environmental variable inhibits species distribution (Xu et al. 2015). Species interactions were analyzed based on residual correlations, where positive correlations between species indicate habitat homogeneity, while negative correlations may suggest habitat heterogeneity.

## 3 Results

### 3.1 MaxEnt Model Accuracy Evaluation

After running the MaxEnt model 10 times for each species, the training AUC values ranged from 0.939 to 0.998, and the testing AUC values ranged from 0.896 to 0.994 (Table S4). According to the AUC evaluation criteria, the model's accuracy in identifying the environmental variables influencing species distributions was rated as “excellent” for all species except the Mongolian gerbil, which was rated as “good.” These results indicate that the MaxEnt model effectively fitted the distribution data of each species, and the predictions are considered reliable. To further assess the threshold‐dependent classification accuracy, the True Skill Statistic (TSS) was calculated. The training TSS values ranged from 0.339 to 0.841, and the testing TSS values ranged from 0.319 to 0.960, suggesting generally good predictive performance (Table S4). Among the seven gerbils, Cheng's Gerbil (Test TSS = 0.960) and Przewalski's Jird (Test TSS = 0.534) exhibited the highest model stability and predictive reliability. In contrast, Great Gerbil and Mongolian Gerbil had relatively lower TSS values (< 0.35), implying higher uncertainty in binary classification, possibly due to their broader ecological niches. Overall, the combination of high AUC and moderate‐to‐high TSS values demonstrates that the MaxEnt models performed well and captured the main environmental suitability patterns of all species.

### 3.2 Major Environmental Variables Influencing Species Distribution

**FIGURE 3:**
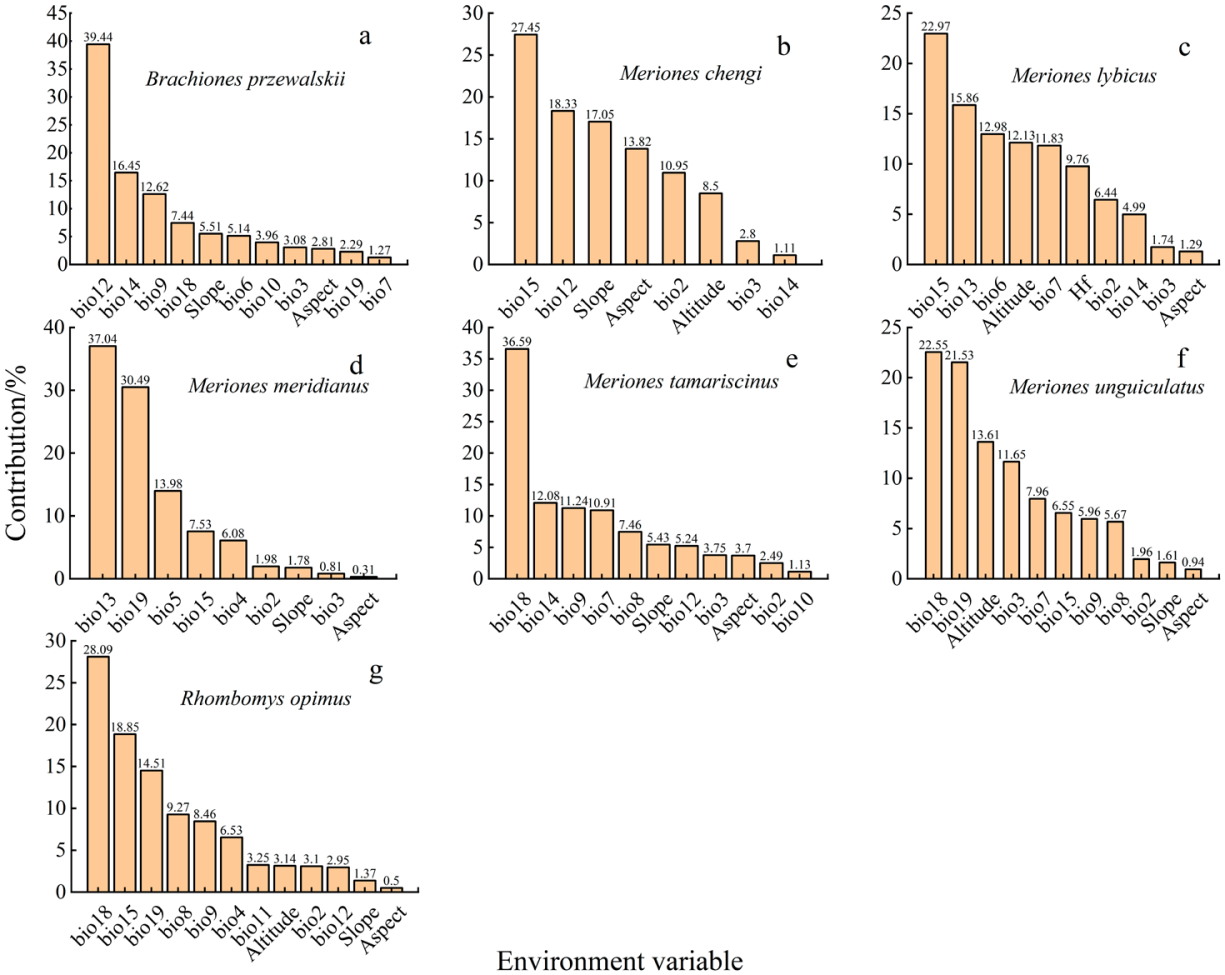
Main environmental variables affecting the distribution of various mouse species. bio2 represents mean diurnal range; bio3 represents isothermality; bio4 represents temperature seasonality; bio5 represents max temperature of warmest month; bio6 represents min temperature of coldest month; bio7 represents temperature annual range; bio8 represents mean temperature of wettest quarter; bio9 represents mean temperature of driest quarter; bio10 represents mean temperature of warmest quarter; bio11 represents mean temperature of coldest quarter; bio12 represents annual precipitation; bio13 represents precipitation of wettest month; bio14 represents precipitation of driest month; bio15 represents precipitation seasonality; bio18 represents precipitation of warmest quarter; bio19 represents precipitation of coldest quarter; Hf represents Human footprint index. The same as below.

The Jackknife test indicated that the main factors influencing gerbil distributions are closely related to temperature and precipitation (Figure 3). Specifically, the distributions of Przewalski's Jird, Tamarisk Gerbil, and Mongolian Gerbil were primarily constrained by precipitation and its seasonal patterns; Cheng's Gerbil and Libyan Jird showed strong dependence on topographic factors, while Mid‐day Gerbil and Great Gerbil were mainly influenced by extreme temperatures and precipitation in cold and warm quarters. Overall, temperature‐ and precipitation‐related variables were the dominant drivers of the potential distributions of these gerbil species.

### 3.3 Species Responses to Environmental Variables

**FIGURE 4:**
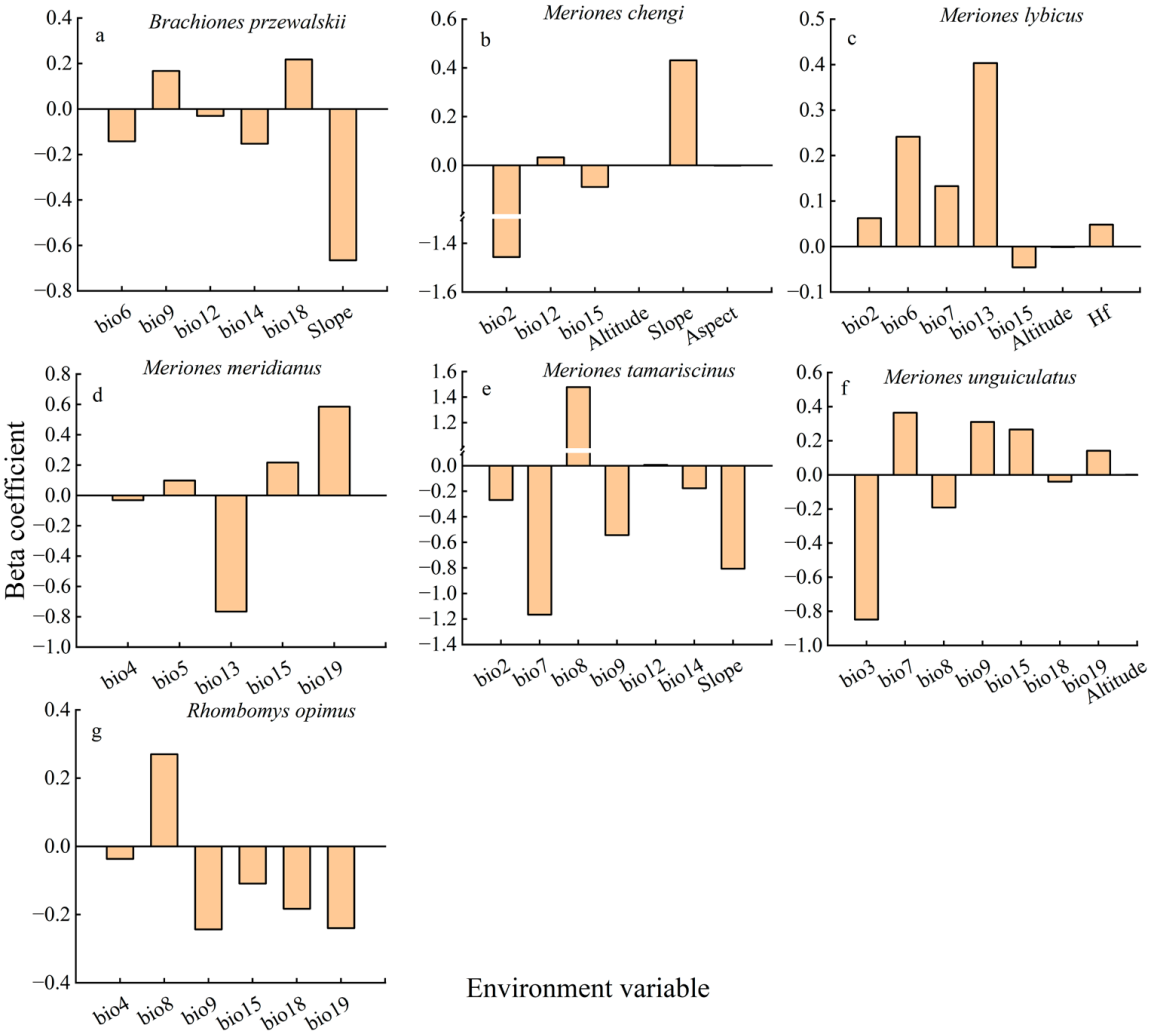
The impact of different environmental variables on species distribution.

Results from the Joint Species Distribution Model (JSDMs) indicated that the distributions of gerbil species were primarily influenced by temperature‐ and precipitation‐related variables, followed by topographic factors and anthropogenic activities (Figure 4). Specifically, some species showed strong dependence on precipitation and its seasonal patterns, while others were more constrained by temperature extremes or topographic factors such as elevation, slope, and aspect. In addition, human activities exerted a certain influence on the distribution of species such as Libyan Jird. Overall, climatic factors remained the dominant determinants of the distribution patterns of these gerbil species.

### 3.4 Analysis of Interspecific Interactions Among Rodents

**FIGURE 5:**
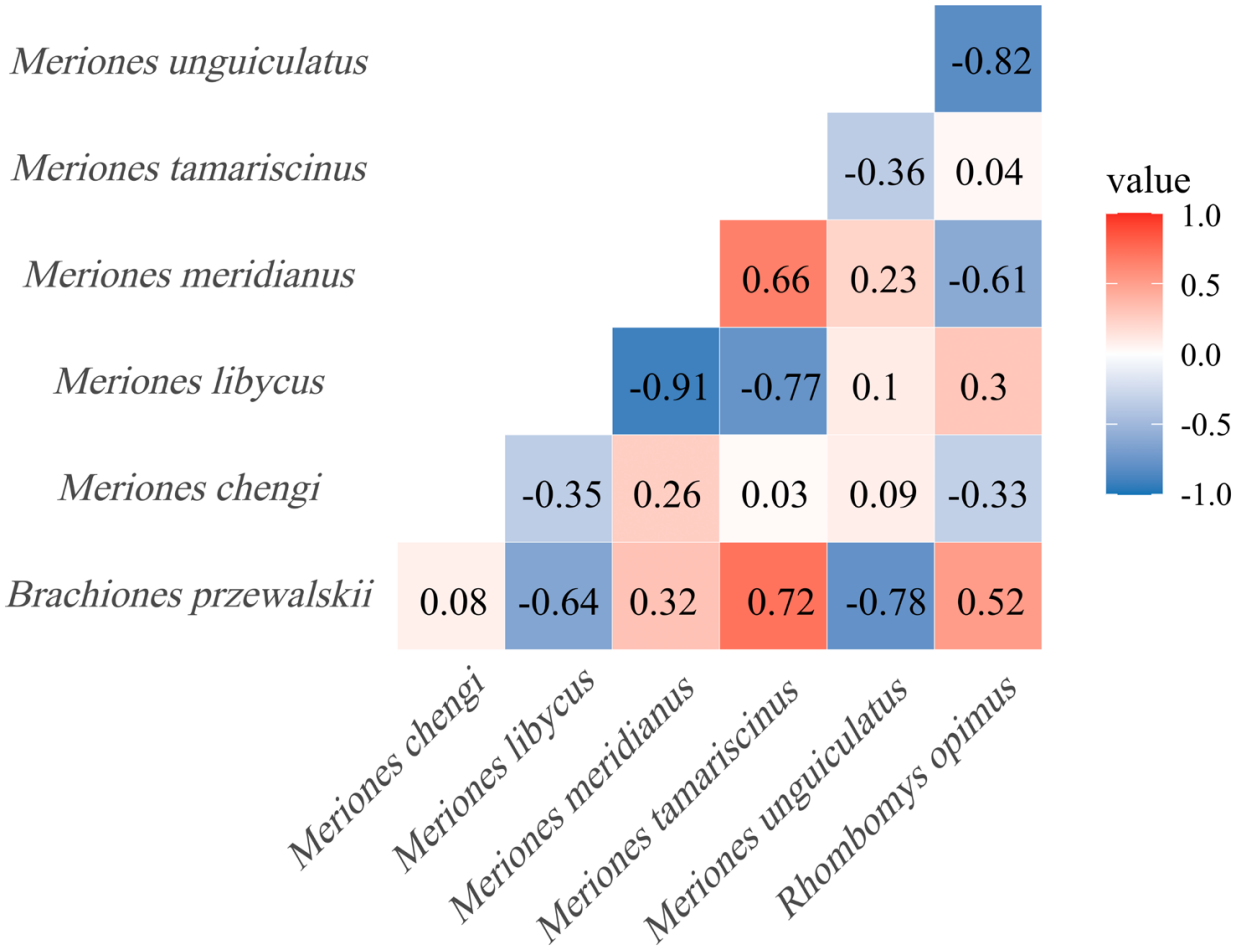
Analysis of Residual Correlation between Species.

After controlling for environmental factors, the residual correlations among species revealed both negative and positive relationships (Figure 5). Strong negative correlations were observed between Mid‐day Gerbil and Libyan Jird, as well as between Mongolian Gerbil and Great Gerbil. In contrast, positive correlations were evident between Tamarisk Gerbil and Przewalski's Jird, and Tamarisk Gerbil and Mid‐day Gerbil. Overall, these results indicate that interspecific coexistence is complex, involving both potential competition and facilitative interactions.

## 4 Discussion

### 4.1 Reliability of Prediction Accuracy in the MaxEnt Model

This study used the MaxEnt model to simulate the distribution patterns of seven gerbil species. The results showed that, except for the Mongolian gerbil, whose testing AUC value was 0.896, classified as “good,” the AUC values for all other species exceeded 0.9, indicating “excellent” predictive performance (Swets 1988). These findings suggest that the selected environmental variables effectively explain and fit the species' distribution patterns, and that the model predictions are highly reliable (Phillips et al. 2006). The slightly lower accuracy for *M. unguiculatus* may be due to its broader geographic distribution and greater ecological adaptability, allowing it to tolerate a wide range of environmental conditions, which complicates precise identification of its optimal habitat. In addition, the spatial representativeness and sample size of field occurrence records may also affect model stability, consistent with the view that “the quality and quantity of occurrence points significantly influence the predictive performance of species distribution models” (Wisz et al. 2013).

### 4.2 Key Environmental Drivers of Species Distribution

Jackknife tests and variable contribution analyses indicated that precipitation‐ and temperature‐related climatic factors are the primary determinants of gerbil species distributions, although species differ markedly in their sensitivity to these factors. The distributions of Przewalski's Jird, Tamarisk Gerbil, and Great Gerbil were more strongly influenced by water‐related variables, including annual precipitation (bio12), precipitation of the driest month (bio14), and precipitation of the warmest quarter (bio18), suggesting that water availability may be a key ecological constraint in arid regions. In contrast, Cheng's Gerbil and Libyan Jird were more affected by precipitation seasonality (bio15), indicating a higher sensitivity to climatic fluctuations. Mid‐day Gerbil and Mongolian Gerbil were primarily influenced by extreme climatic variables, such as precipitation of the coldest quarter (bio19) and maximum temperature of the warmest month (bio5), implying that their distributions may be limited by seasonal extreme events. These differences reflect niche differentiation among species (Wang et al. 2020). As emphasized by niche hypervolume theory, species distributions are shaped by multiple environmental dimensions, and interspecific differences in environmental tolerance underlie niche differentiation and coexistence (Lu et al. 2021). In this study, elevation was included as an environmental variable. While elevation is often considered a proxy for climatic factors in species distribution models rather than a direct driver, it can indirectly influence species distributions by affecting temperature, precipitation, soil, and vegetation patterns (Hof et al. 2012). In the study region, topographic variation significantly shapes local climate and habitat heterogeneity, making the inclusion of elevation ecologically meaningful (Kunwar et al. 2024). Although human activities such as grazing are often considered important drivers of habitat availability and species interactions in arid and semi‐arid ecosystems, our analysis did not detect a significant effect of grazing intensity on the distribution patterns of the studied gerbil species. This result is consistent with previous studies showing that, in some semi‐arid systems, grazing primarily influences vegetation cover or biomass rather than directly altering species richness or distribution (Tulloch et al. 2023; Arroyo et al. 2024; Wu, Goldfeld, et al. 2024; Wu, Li, et al. 2024).

### 4.3 Variation in Species Responses to Environmental Variables

The beta coefficients from the Joint Species Distribution Models (JSDMs) further revealed species‐specific responses to environmental variables. The occurrence probabilities of Przewalski's Jird and Mid‐day Gerbil increased with improved water availability or rising temperatures, whereas Tamarisk Gerbil and Great Gerbil exhibited negative responses, indicating differing adaptive strategies to changes in hydrothermal conditions. These differences likely reflect ecological niche differentiation: on one hand, species have evolved distinct physiological and behavioral adaptations to arid and semi‐arid environments over long‐term evolutionary processes; on the other hand, improvements in hydrothermal conditions may alter interspecific competitive relationships, indirectly influencing distribution patterns (Wang 1983). This pattern aligns with the concept that climatic factors determine the potential distribution range at broad spatial scales, while biotic interactions and ecological adaptations further modulate the realized distributions of species (Wisz et al. 2013).

### 4.4 Interspecific Interactions and Mechanisms of Coexistence

The species correlation matrix estimated by the Joint Species Distribution Models (JSDMs) revealed interspecific interactions. Such interactions are central to ecological community functioning and may be key drivers of species distributions. Species can directly influence one another through mechanisms such as competition, facilitation, and predation, with the strength and outcome of these interactions determining coexistence potential (Dulude‐de Broin et al. 2023). Residual correlation analysis, conducted after controlling for environmental factors, showed that many species pairs exhibited significant negative correlations, indicating interspecific exclusion. Strong negative residual correlations between Mid‐day Gerbil and Libyan Jird, as well as between Mongolian Gerbil and Great Gerbil, likely reflect competition for food resources or burrow sites. In areas with similar hydrothermal conditions, resource overlap may intensify competition, leading to mutually exclusive distribution patterns. Conversely, positive correlations between Tamarisk Gerbil and Przewalski's Jird, and between Tamarisk Gerbil and Mid‐day Gerbil, suggest that these species may coexist through spatial or temporal niche differentiation, and in some cases may even exhibit facilitative interactions. Differences in diel activity patterns or feeding preferences between Great Gerbil and Mid‐day Gerbil may further reduce direct competition (Wang 1983). These findings are consistent with the JSDMs framework, which posits that residual correlations after accounting for environmental factors can reveal biotic interactions beyond environmental influences, providing insights into community assembly mechanisms (Warton et al. 2015). Therefore, the observed negative and positive correlations not only reflect potential competition and facilitation but also support the concept of a “dual mechanism” whereby both environmental adaptation and interspecific interactions jointly shape community structure.

## 5 Conclusion

This study focused on gerbil rodent communities, integrating species distribution data with environmental variables. We employed the maximum entropy model (MaxEnt) to identify the key environmental drivers of species distributions and combined it with joint species distribution models (JSDMs) to analyze interspecific relationships and the influence of environmental factors.

1. The accuracy evaluation demonstrated that the MaxEnt models achieved high discrimination and predictive reliability, with AUC values above 0.89 and TSS values mostly exceeding 0.4. Overall, these results indicate that the MaxEnt models effectively simulated the potential distributions and environmental suitability of all gerbil species.
2. The relative importance of environmental variables varied markedly among species. Overall, precipitation and temperature were the dominant factors shaping species distributions. Topographic factors and human activities also affected distributions at the local scale, while grazing intensity showed no significant influence.
3. Species exhibited selective responses to environmental variables. For some species, distribution was promoted by precipitation seasonality, extreme temperatures, and quarterly climatic factors, whereas others showed negative responses. This reflects interspecific differences in ecological niche utilization among gerbils.
4. The residual correlation analysis revealed several negative associations, suggesting strong competitive exclusion among certain species. In contrast, some positive correlations were observed, indicating potential co‐distribution or resource complementarity.

In summary, the distribution patterns of gerbil rodents in arid desert regions are driven not only by climatic and topographic factors but also by complex interspecific interactions. These findings enhance our understanding of coexistence mechanisms in desert rodent communities and provide scientific support for regional ecosystem conservation and rodent pest management. Nevertheless, this study is limited by the availability of species distribution data and the selection of environmental variables, and interpretations of interspecific relationships largely rely on model inference. Future research should incorporate higher‐resolution monitoring data and field‐based empirical studies to more comprehensively reveal the distribution patterns and coexistence mechanisms of gerbil rodents.

## Author Contributions

**Jiuqi Zhao:** conceptualization (equal), data curation (equal), formal analysis (equal), investigation (equal), methodology (equal), resources (equal), software (equal), visualization (equal), writing – original draft (equal). **Rong Zhang:** conceptualization (equal), data curation (equal), formal analysis (equal), investigation (equal), methodology (equal), resources (equal), software (equal), visualization (equal), writing – original draft (equal). **Yiqiang Dong:** funding acquisition (equal). **Mengyue Wang:** visualization (equal). **Wenshan Chen:** visualization (equal). **Die Chen:** visualization (equal). **Suwen Yang:** investigation (equal), methodology (equal), project administration (equal), resources (equal), software (equal), supervision (equal), writing – review and editing (equal).

## Conflicts of Interest

The authors declare no conflicts of interest.

## Supporting information


**Table S1:** Species occurrence points used in Maxent modeling.
**Table S2:** Species occurrence points used in JSDM modeling.

**Data S1:** ece372468‐sup‐0002‐Supinfo.docx.

## Data Availability

All the data supporting the findings of this study are available in the Supporting Information files.
