# Supplementary material for: Environmental Limitations and Interspecific Interactions Across the Distribution Range of Gerbils
Source: Ecol Evol. 2025 Nov 10;15(11):e72468. doi: 10.1002/ece3.72468 (PMC12602260; doi:10.1002/ece3.72468)
Supplement: Supplementary file 1 — Table S1: Species occurrence points used in Maxent modeling. Table S2: Species occurrence points used in JSDM modeling. [file ECE3-15-e72468-s002.docx]

**Table S1 Species occurrence points used in Maxent modeling**

| Przewalski’s Jird(*Brachiones przewalskii*) | | Cheng’s Gerbil(*Meriones chengi*) | | Libyan Jird(*Meriones libycus*) | | Mid-day Gerbil(*Meriones meridianus*) | | Tamarisk Gerbil(*Meriones tamariscinus*) | | Mongolian Gerbil(*Meriones unguiculatus*) | | Great Gerbil(*Rhombomys opimus*) | |
| --- | --- | --- | --- | --- | --- | --- | --- | --- | --- | --- | --- | --- | --- |
| longitude | latitude | longitude | latitude | longitude | latitude | longitude | latitude | longitude | latitude | longitude | latitude | longitude | latitude |
| 77.1125 | 39.5625 | 89.09583333 | 42.90416667 | 104.9958333 | 37.50416667 | 100.0958333 | 43.60416667 | 70.30416667 | 46.10416667 | 100.3958333 | 43.50416667 | 100.0208333 | 38.30416667 |
| 77.19583333 | 38.40416667 | 80.39583333 | 44.20416667 | 55.10416667 | 45.90416667 | 100.1875 | 38.64583333 | 73.00416667 | 44.00416667 | 100.3958333 | 44.90416667 | 100.0958333 | 45.20416667 |
| 77.47083333 | 37.65416667 | 88.77916667 | 43.0875 | 73.00416667 | 43.00416667 | 100.3958333 | 46.00416667 | 73.99583333 | 46.40416667 | 101.4958333 | 45.20416667 | 101.0458333 | 42.27083333 |
| 77.7875 | 38.6625 | 87.8375 | 43.52083333 | 73.00416667 | 44.00416667 | 100.4541667 | 38.72083333 | 79.09583333 | 45.10416667 | 101.8958333 | 45.20416667 | 101.4958333 | 38.34583333 |
| 79.60416667 | 37.5625 | 88.07083333 | 43.5625 | 74.99583333 | 44.00416667 | 100.4958333 | 43.70416667 | 80.69583333 | 44.10416667 | 101.9958333 | 43.90416667 | 101.4958333 | 45.20416667 |
| 79.99583333 | 37.30416667 | 75.94583333 | 39.85416667 | 74.99583333 | 45.00416667 | 100.5875 | 42.2125 | 80.79583333 | 44.10416667 | 101.9958333 | 44.70416667 | 101.9958333 | 43.90416667 |
| 80.2875 | 37.1875 | 76.1875 | 40.10416667 | 75.39583333 | 43.90416667 | 100.6708333 | 38.7625 | 82.19583333 | 46.40416667 | 101.9958333 | 45.40416667 | 102.2125 | 41.22083333 |
| 80.3625 | 37.27083333 | 76.0375 | 40.1125 | 75.94583333 | 39.85416667 | 100.6958333 | 45.10416667 | 84.6625 | 45.19583333 | 102.2958333 | 43.80416667 | 102.2958333 | 43.80416667 |
| 81.0125 | 36.3125 | 76.74583333 | 40.15416667 | 76.0375 | 40.1125 | 100.8291667 | 42.19583333 | 84.80416667 | 41.85416667 | 102.5958333 | 46.10416667 | 102.9125 | 38.42083333 |
| 81.37916667 | 37.60416667 | 55.10416667 | 45.90416667 | 76.1875 | 40.10416667 | 100.8791667 | 38.6875 | 85.09583333 | 47.90416667 | 102.6958333 | 45.40416667 | 103.0208333 | 38.05416667 |
| 81.3875 | 37.39583333 |  |  | 76.74583333 | 40.15416667 | 101.0041667 | 38.6125 | 86.35416667 | 44.57916667 | 103.2958333 | 43.80416667 | 103.4958333 | 42.80416667 |
| 81.42083333 | 37.32916667 |  |  | 76.99583333 | 44.00416667 | 101.2625 | 38.5875 | 87.2375 | 44.3375 | 103.6958333 | 47.40416667 | 103.6958333 | 44.20416667 |
| 81.4625 | 37.24583333 |  |  | 76.99583333 | 45.00416667 | 101.4958333 | 45.20416667 | 87.49583333 | 47.10416667 | 103.7958333 | 43.60416667 | 104.0958333 | 44.60416667 |
| 81.49583333 | 37.0625 |  |  | 77.1125 | 39.5625 | 101.5708333 | 38.5625 | 87.8125 | 45.70416667 | 103.7958333 | 45.50416667 | 105.1208333 | 39.57916667 |
| 81.5125 | 37.1875 |  |  | 77.99583333 | 46.00416667 | 101.8708333 | 38.5875 | 88.2875 | 47.74583333 | 103.9958333 | 43.50416667 | 105.4125 | 39.5375 |
| 81.54583333 | 37.1125 |  |  | 78.99583333 | 43.00416667 | 101.9958333 | 43.90416667 | 88.4875 | 42.29583333 | 103.9958333 | 43.60416667 | 105.4208333 | 39.5375 |
| 82.8125 | 37.34583333 |  |  | 78.99583333 | 44.00416667 | 101.9958333 | 44.70416667 | 88.87083333 | 43.97083333 | 103.9958333 | 45.40416667 | 105.5458333 | 40.07083333 |
| 82.8875 | 37.62916667 |  |  | 80.8875 | 44.0875 | 102.0625 | 38.7125 | 88.99583333 | 44.19583333 | 104.0958333 | 43.50416667 | 105.6625 | 38.8375 |
| 83.3375 | 37.90416667 |  |  | 80.9625 | 44.12916667 | 102.0958333 | 44.60416667 | 89.10416667 | 44.12083333 | 104.0958333 | 47.90416667 | 105.7708333 | 39.6375 |
| 83.47916667 | 37.9125 |  |  | 81.22916667 | 44.12083333 | 102.2958333 | 43.80416667 | 91.09583333 | 45.30416667 | 104.2958333 | 47.80416667 | 106.1458333 | 41.5125 |
| 85.49583333 | 38.30416667 |  |  | 81.22916667 | 44.12916667 | 102.3958333 | 43.80416667 | 91.09583333 | 45.70416667 | 104.5958333 | 47.10416667 | 106.1791667 | 39.47916667 |
| 87.54583333 | 38.5625 |  |  | 82.27916667 | 44.5375 | 102.4125 | 38.84583333 | 91.09583333 | 46.10416667 | 104.9958333 | 37.50416667 | 107.0708333 | 41.0875 |
|  |  |  |  | 82.29583333 | 45.00416667 | 102.8791667 | 38.97916667 | 91.19583333 | 44.39583333 | 104.9958333 | 47.70416667 | 108.4625 | 42.07083333 |
|  |  |  |  | 82.29583333 | 45.10416667 | 103.2625 | 39.12083333 | 92.19583333 | 45.50416667 | 105.1958333 | 47.90416667 | 108.5125 | 41.5875 |
|  |  |  |  | 82.40416667 | 45.5625 | 103.4041667 | 38.5125 | 93.29583333 | 44.30416667 | 105.1958333 | 49.20416667 | 108.6541667 | 40.7375 |
|  |  |  |  | 82.42083333 | 45.5625 | 103.5958333 | 47.60416667 | 93.39583333 | 44.10416667 | 105.2958333 | 47.90416667 | 109.9958333 | 42.2375 |
|  |  |  |  | 82.49583333 | 45.00416667 | 103.6125 | 39.22083333 | 94.39583333 | 45.60416667 | 105.3958333 | 45.00416667 | 110.3875 | 42.2375 |
|  |  |  |  | 82.89583333 | 44.60416667 | 103.6958333 | 47.40416667 |  |  | 105.6958333 | 47.20416667 | 110.4291667 | 41.69583333 |
|  |  |  |  | 83.04583333 | 44.62083333 | 103.8958333 | 39.22916667 |  |  | 105.7958333 | 47.80416667 | 110.6375 | 42.39583333 |
|  |  |  |  | 84.30416667 | 41.7375 | 103.9958333 | 43.50416667 |  |  | 105.8958333 | 46.90416667 | 111.0958333 | 42.50416667 |
|  |  |  |  | 84.74583333 | 44.19583333 | 104.0958333 | 43.50416667 |  |  | 105.8958333 | 47.70416667 | 111.7041667 | 41.5375 |
|  |  |  |  | 84.9875 | 44.77916667 | 104.1541667 | 38.3625 |  |  | 105.8958333 | 47.80416667 | 50.70416667 | 44.50416667 |
|  |  |  |  | 85.3375 | 46.84583333 | 104.2625 | 36.2125 |  |  | 105.9958333 | 47.40416667 | 51.10416667 | 44.40416667 |
|  |  |  |  | 85.3625 | 44.1125 | 104.2958333 | 47.80416667 |  |  | 105.9958333 | 47.90416667 | 51.90416667 | 48.50416667 |
|  |  |  |  | 85.42916667 | 41.84583333 | 104.4208333 | 39.27083333 |  |  | 106.1958333 | 50.20416667 | 52.00416667 | 44.00416667 |
|  |  |  |  | 85.57916667 | 46.84583333 | 104.4375 | 37.9625 |  |  | 106.2958333 | 46.90416667 | 52.10416667 | 44.20416667 |
|  |  |  |  | 85.6875 | 46.8125 | 104.4791667 | 39.65416667 |  |  | 106.2958333 | 47.60416667 | 52.20416667 | 44.10416667 |
|  |  |  |  | 85.87083333 | 44.1375 | 104.5041667 | 40.17916667 |  |  | 106.3958333 | 46.10416667 | 53.20416667 | 44.10416667 |
|  |  |  |  | 85.89583333 | 44.05416667 | 104.5458333 | 40.57916667 |  |  | 106.6958333 | 47.90416667 | 53.70416667 | 47.10416667 |
|  |  |  |  | 85.97083333 | 44.72083333 | 104.6791667 | 41.42916667 |  |  | 106.6958333 | 48.10416667 | 53.80416667 | 47.10416667 |
|  |  |  |  | 86.02916667 | 41.7625 | 104.7208333 | 38.54583333 |  |  | 106.7541667 | 38.2875 | 54.40416667 | 47.30416667 |
|  |  |  |  | 86.02916667 | 44.0125 | 104.7458333 | 41.0625 |  |  | 106.7958333 | 44.80416667 | 55.60416667 | 45.90416667 |
|  |  |  |  | 86.10416667 | 46.8375 | 104.8291667 | 37.77083333 |  |  | 106.9041667 | 38.62083333 | 56.30416667 | 38.40416667 |
|  |  |  |  | 86.17083333 | 46.7375 | 104.8291667 | 39.1625 |  |  | 107.0458333 | 38.3375 | 56.30416667 | 39.00416667 |
|  |  |  |  | 86.22083333 | 44.4625 | 105.1208333 | 38.49583333 |  |  | 107.0625 | 38.6375 | 58.20416667 | 43.20416667 |
|  |  |  |  | 86.2375 | 44.52083333 | 105.2958333 | 42.20416667 |  |  | 107.1291667 | 38.54583333 | 58.40416667 | 40.30416667 |
|  |  |  |  | 86.2625 | 46.7875 | 105.2958333 | 42.50416667 |  |  | 107.2041667 | 38.87916667 | 61.30416667 | 47.80416667 |
|  |  |  |  | 86.3125 | 46.8375 | 105.3625 | 38.3375 |  |  | 107.2958333 | 45.80416667 | 61.70416667 | 46.80416667 |
|  |  |  |  | 86.35416667 | 44.57916667 | 106.7958333 | 42.50416667 |  |  | 107.4041667 | 38.3625 | 62.30416667 | 35.30416667 |
|  |  |  |  | 86.42083333 | 46.9125 | 106.7958333 | 44.80416667 |  |  | 107.4458333 | 38.65416667 | 63.20416667 | 38.60416667 |
|  |  |  |  | 86.5375 | 44.8625 | 107.1958333 | 43.00416667 |  |  | 107.5375 | 38.12083333 | 63.30416667 | 45.60416667 |
|  |  |  |  | 86.5375 | 46.9875 | 107.2958333 | 43.00416667 |  |  | 107.6625 | 37.87083333 | 63.40416667 | 40.20416667 |
|  |  |  |  | 86.65416667 | 44.3375 | 107.2958333 | 45.80416667 |  |  | 107.8958333 | 39.20416667 | 63.50416667 | 45.60416667 |
|  |  |  |  | 86.9875 | 44.72916667 | 108.9958333 | 43.10416667 |  |  | 107.9541667 | 38.3125 | 63.60416667 | 40.10416667 |
|  |  |  |  | 87.0125 | 43.54583333 | 109.4875 | 36.79583333 |  |  | 108.1458333 | 38.1125 | 64.60416667 | 40.60416667 |
|  |  |  |  | 87.02083333 | 44.80416667 | 109.4958333 | 36.60416667 |  |  | 108.3458333 | 38.1625 | 64.70416667 | 38.90416667 |
|  |  |  |  | 87.0375 | 44.7375 | 111.0958333 | 42.50416667 |  |  | 108.3708333 | 37.64583333 | 64.70416667 | 39.60416667 |
|  |  |  |  | 87.0375 | 44.74583333 | 111.8375 | 43.14583333 |  |  | 108.3958333 | 47.60416667 | 65.10416667 | 42.00416667 |
|  |  |  |  | 87.0375 | 44.75416667 | 111.9958333 | 43.80416667 |  |  | 108.6791667 | 38.1875 | 66.10416667 | 41.00416667 |
|  |  |  |  | 87.1875 | 43.72916667 | 112.2541667 | 39.07916667 |  |  | 109.4958333 | 34.70416667 | 66.20416667 | 41.40416667 |
|  |  |  |  | 87.29583333 | 44.8375 | 112.2958333 | 39.00416667 |  |  | 110.6958333 | 40.60416667 | 66.50416667 | 44.10416667 |
|  |  |  |  | 87.30416667 | 44.77916667 | 112.3458333 | 43.64583333 |  |  | 111.6958333 | 40.80416667 | 67.30416667 | 40.70416667 |
|  |  |  |  | 87.30416667 | 44.80416667 | 112.4125 | 38.57916667 |  |  | 112.0958333 | 45.30416667 | 68.30416667 | 45.00416667 |
|  |  |  |  | 87.3125 | 44.4125 | 112.5958333 | 37.90416667 |  |  | 112.2958333 | 37.70416667 | 69.60416667 | 42.30416667 |
|  |  |  |  | 87.35416667 | 44.52083333 | 113.2708333 | 43.97916667 |  |  | 112.6791667 | 43.12083333 | 71.60416667 | 41.10416667 |
|  |  |  |  | 87.4125 | 44.67083333 | 115.9291667 | 42.0625 |  |  | 112.7208333 | 44.5125 | 73.49583333 | 44.90416667 |
|  |  |  |  | 87.45416667 | 44.62916667 | 65.02083333 | 43.52083333 |  |  | 112.7625 | 43.94583333 | 74.39583333 | 44.40416667 |
|  |  |  |  | 87.47083333 | 44.62083333 | 74.5875 | 39.8375 |  |  | 112.7625 | 44.35416667 | 74.49583333 | 44.40416667 |
|  |  |  |  | 87.49583333 | 44.6375 | 75.0375 | 39.47083333 |  |  | 112.7958333 | 42.50416667 | 74.89583333 | 45.90416667 |
|  |  |  |  | 87.5625 | 46.47083333 | 75.07083333 | 39.39583333 |  |  | 112.8291667 | 42.87916667 | 74.99583333 | 44.00416667 |
|  |  |  |  | 87.59583333 | 45.77083333 | 75.07916667 | 39.27083333 |  |  | 112.9958333 | 46.20416667 | 74.99583333 | 46.20416667 |
|  |  |  |  | 87.74583333 | 46.22916667 | 75.37083333 | 39.34583333 |  |  | 113.3125 | 42.50416667 | 75.19583333 | 45.50416667 |
|  |  |  |  | 87.74583333 | 46.50416667 | 75.37083333 | 39.69583333 |  |  | 113.4458333 | 41.90416667 | 75.39583333 | 44.40416667 |
|  |  |  |  | 87.7625 | 45.6125 | 75.49583333 | 39.5625 |  |  | 116.4958333 | 46.80416667 | 75.49583333 | 44.30416667 |
|  |  |  |  | 87.7875 | 45.69583333 | 75.49583333 | 40.50416667 |  |  | 117.4958333 | 49.00416667 | 75.99583333 | 44.90416667 |
|  |  |  |  | 87.9125 | 46.5125 | 75.5125 | 39.25416667 |  |  | 117.6958333 | 49.00416667 | 76.89583333 | 44.20416667 |
|  |  |  |  | 87.9375 | 46.45416667 | 75.5875 | 39.55416667 |  |  | 73.00416667 | 43.00416667 | 76.99583333 | 44.10416667 |
|  |  |  |  | 87.94583333 | 46.52083333 | 75.6375 | 39.29583333 |  |  | 74.99583333 | 44.00416667 | 76.99583333 | 44.30416667 |
|  |  |  |  | 87.97916667 | 46.27916667 | 75.75416667 | 39.8875 |  |  | 74.99583333 | 45.00416667 | 77.09583333 | 43.90416667 |
|  |  |  |  | 88.22916667 | 42.2375 | 75.9875 | 39.80416667 |  |  | 75.99583333 | 44.00416667 | 78.29583333 | 43.30416667 |
|  |  |  |  | 88.29583333 | 44.52083333 | 76.09583333 | 39.80416667 |  |  | 76.99583333 | 45.00416667 | 78.49583333 | 43.30416667 |
|  |  |  |  | 88.3125 | 42.14583333 | 76.1375 | 39.12916667 |  |  | 77.99583333 | 46.00416667 | 78.49583333 | 43.50416667 |
|  |  |  |  | 88.32083333 | 42.3875 | 76.15416667 | 39.07083333 |  |  | 78.99583333 | 43.00416667 | 78.59583333 | 43.40416667 |
|  |  |  |  | 88.35416667 | 42.09583333 | 76.20416667 | 39.1125 |  |  | 78.99583333 | 44.00416667 | 78.59583333 | 43.90416667 |
|  |  |  |  | 88.3625 | 42.34583333 | 76.22916667 | 38.70416667 |  |  | 79.99583333 | 45.00416667 | 78.79583333 | 43.40416667 |
|  |  |  |  | 88.37083333 | 42.29583333 | 76.2375 | 39.5375 |  |  | 89.99583333 | 48.90416667 | 78.79583333 | 44.00416667 |
|  |  |  |  | 88.5125 | 42.3125 | 76.24583333 | 39.57083333 |  |  | 92.29583333 | 47.50416667 | 78.89583333 | 43.40416667 |
|  |  |  |  | 88.57916667 | 42.37083333 | 76.24583333 | 39.8375 |  |  | 92.29583333 | 47.80416667 | 78.89583333 | 44.00416667 |
|  |  |  |  | 88.6625 | 42.37083333 | 76.27916667 | 39.8375 |  |  | 92.39583333 | 47.70416667 | 78.99583333 | 43.30416667 |
|  |  |  |  | 88.77083333 | 44.25416667 | 76.2875 | 39.12916667 |  |  | 92.49583333 | 47.70416667 | 78.99583333 | 43.40416667 |
|  |  |  |  | 88.77083333 | 44.32916667 | 76.29583333 | 39.47083333 |  |  | 92.79583333 | 47.40416667 | 78.99583333 | 43.50416667 |
|  |  |  |  | 88.79583333 | 42.3375 | 76.32916667 | 39.17083333 |  |  | 92.79583333 | 47.70416667 | 79.09583333 | 43.30416667 |
|  |  |  |  | 88.82083333 | 42.34583333 | 76.3875 | 39.79583333 |  |  | 92.89583333 | 47.40416667 | 79.09583333 | 43.40416667 |
|  |  |  |  | 88.84583333 | 42.8625 | 76.39583333 | 39.12083333 |  |  | 93.19583333 | 48.10416667 | 79.19583333 | 44.00416667 |
|  |  |  |  | 88.84583333 | 43.2125 | 76.4125 | 39.79583333 |  |  | 93.39583333 | 46.40416667 | 79.29583333 | 43.50416667 |
|  |  |  |  | 88.87916667 | 42.3375 | 76.45416667 | 39.0875 |  |  | 93.59583333 | 45.30416667 | 79.39583333 | 44.20416667 |
|  |  |  |  | 88.94583333 | 42.87083333 | 76.47083333 | 39.50416667 |  |  | 93.59583333 | 45.50416667 | 79.49583333 | 44.20416667 |
|  |  |  |  | 88.95416667 | 42.87083333 | 76.5375 | 39.07916667 |  |  | 93.69583333 | 45.50416667 | 80.49583333 | 44.00416667 |
|  |  |  |  | 88.97916667 | 43.87916667 | 76.57916667 | 39.10416667 |  |  | 93.69583333 | 45.60416667 | 81.69583333 | 44.6125 |
|  |  |  |  | 88.99583333 | 42.87916667 | 76.57916667 | 39.12916667 |  |  | 94.49583333 | 46.10416667 | 81.90416667 | 44.62916667 |
|  |  |  |  | 89.0125 | 42.90416667 | 76.60416667 | 39.02916667 |  |  | 94.79583333 | 40.20416667 | 82.0375 | 44.6625 |
|  |  |  |  | 89.02916667 | 42.8125 | 76.62083333 | 39.84583333 |  |  | 94.89583333 | 49.00416667 | 82.0375 | 44.8125 |
|  |  |  |  | 89.0375 | 42.85416667 | 76.62083333 | 39.85416667 |  |  | 95.09583333 | 46.20416667 | 82.05416667 | 44.67083333 |
|  |  |  |  | 89.09583333 | 42.90416667 | 76.62916667 | 37.72083333 |  |  | 95.29583333 | 46.30416667 | 82.05416667 | 44.7875 |
|  |  |  |  | 89.12083333 | 44.7125 | 76.62916667 | 39.0125 |  |  | 96.09583333 | 46.40416667 | 82.07083333 | 44.7625 |
|  |  |  |  | 89.12916667 | 43.94583333 | 76.62916667 | 39.14583333 |  |  | 96.09583333 | 48.30416667 | 82.0875 | 44.6875 |
|  |  |  |  | 89.3375 | 45.14583333 | 76.6375 | 38.65416667 |  |  | 96.39583333 | 45.70416667 | 82.0875 | 44.69583333 |
|  |  |  |  | 89.7875 | 42.57916667 | 76.6625 | 39.10416667 |  |  | 96.39583333 | 46.20416667 | 82.25416667 | 44.3875 |
|  |  |  |  | 89.8125 | 38.15416667 | 76.80416667 | 39.3875 |  |  | 96.39583333 | 46.30416667 | 82.29583333 | 45.00416667 |
|  |  |  |  | 89.8625 | 42.60416667 | 76.8375 | 39.5125 |  |  | 96.79583333 | 44.90416667 | 82.29583333 | 45.10416667 |
|  |  |  |  | 90.0375 | 38.37083333 | 76.92916667 | 37.3625 |  |  | 96.79583333 | 47.80416667 | 82.3375 | 44.47083333 |
|  |  |  |  | 90.20416667 | 45.64583333 | 76.92916667 | 37.99583333 |  |  | 99.19583333 | 45.70416667 | 82.3625 | 45.62916667 |
|  |  |  |  | 90.22916667 | 45.0375 | 76.9375 | 40.2125 |  |  | 99.19583333 | 46.10416667 | 82.37916667 | 44.92083333 |
|  |  |  |  | 90.2375 | 45.0125 | 76.99583333 | 38.4625 |  |  | 99.29583333 | 46.10416667 | 82.3875 | 44.90416667 |
|  |  |  |  | 90.27916667 | 45.12083333 | 77.0125 | 38.02083333 |  |  |  |  | 82.39583333 | 45.6375 |
|  |  |  |  | 90.27916667 | 45.1625 | 77.0125 | 38.05416667 |  |  |  |  | 82.40416667 | 45.5625 |
|  |  |  |  | 90.32083333 | 45.20416667 | 77.0625 | 39.59583333 |  |  |  |  | 82.42083333 | 45.5625 |
|  |  |  |  | 90.3625 | 45.2125 | 77.07916667 | 38.5375 |  |  |  |  | 82.45416667 | 44.90416667 |
|  |  |  |  | 90.37916667 | 45.27083333 | 77.0875 | 38.0375 |  |  |  |  | 82.47083333 | 45.52083333 |
|  |  |  |  | 90.49583333 | 45.17083333 | 77.0875 | 38.5875 |  |  |  |  | 82.4875 | 44.42083333 |
|  |  |  |  | 90.55416667 | 44.24583333 | 77.10416667 | 37.52083333 |  |  |  |  | 82.49583333 | 45.00416667 |
|  |  |  |  | 90.57083333 | 44.25416667 | 77.1125 | 39.5625 |  |  |  |  | 82.50416667 | 44.52083333 |
|  |  |  |  | 91.2625 | 44.4125 | 77.1375 | 37.29583333 |  |  |  |  | 82.5125 | 44.50416667 |
|  |  |  |  | 93.02083333 | 42.90416667 | 77.1625 | 38.02083333 |  |  |  |  | 82.54583333 | 45.47916667 |
|  |  |  |  | 93.5875 | 43.07083333 | 77.17916667 | 40.25416667 |  |  |  |  | 82.57083333 | 45.55416667 |
|  |  |  |  | 94.84583333 | 41.92083333 | 77.19583333 | 37.40416667 |  |  |  |  | 82.5875 | 45.45416667 |
|  |  |  |  | 95.67083333 | 42.19583333 | 77.19583333 | 37.55416667 |  |  |  |  | 82.60416667 | 45.19583333 |
|  |  |  |  |  |  | 77.2125 | 39.67083333 |  |  |  |  | 82.6125 | 45.4125 |
|  |  |  |  |  |  | 77.24583333 | 37.4375 |  |  |  |  | 82.6125 | 45.4375 |
|  |  |  |  |  |  | 77.25416667 | 37.4875 |  |  |  |  | 82.62916667 | 45.2125 |
|  |  |  |  |  |  | 77.2625 | 38.72083333 |  |  |  |  | 82.62916667 | 45.27916667 |
|  |  |  |  |  |  | 77.2625 | 40.29583333 |  |  |  |  | 82.62916667 | 45.37083333 |
|  |  |  |  |  |  | 77.27083333 | 37.4625 |  |  |  |  | 82.6375 | 44.5125 |
|  |  |  |  |  |  | 77.27916667 | 38.80416667 |  |  |  |  | 82.64583333 | 44.92916667 |
|  |  |  |  |  |  | 77.3125 | 37.52916667 |  |  |  |  | 82.6625 | 45.15416667 |
|  |  |  |  |  |  | 77.3125 | 38.77083333 |  |  |  |  | 82.67916667 | 45.40416667 |
|  |  |  |  |  |  | 77.3125 | 39.6875 |  |  |  |  | 82.67916667 | 45.4125 |
|  |  |  |  |  |  | 77.32916667 | 37.57916667 |  |  |  |  | 82.69583333 | 44.32916667 |
|  |  |  |  |  |  | 77.3625 | 37.62916667 |  |  |  |  | 82.7375 | 44.2375 |
|  |  |  |  |  |  | 77.40416667 | 37.67083333 |  |  |  |  | 82.74583333 | 45.1125 |
|  |  |  |  |  |  | 77.4125 | 37.72083333 |  |  |  |  | 82.75416667 | 40.9625 |
|  |  |  |  |  |  | 77.44583333 | 37.6125 |  |  |  |  | 82.77083333 | 44.37083333 |
|  |  |  |  |  |  | 77.4625 | 37.42916667 |  |  |  |  | 82.77916667 | 44.55416667 |
|  |  |  |  |  |  | 77.4625 | 37.75416667 |  |  |  |  | 82.7875 | 44.39583333 |
|  |  |  |  |  |  | 77.47083333 | 37.65416667 |  |  |  |  | 82.80416667 | 44.27916667 |
|  |  |  |  |  |  | 77.47083333 | 39.70416667 |  |  |  |  | 82.8125 | 37.34583333 |
|  |  |  |  |  |  | 77.4875 | 37.6875 |  |  |  |  | 82.8125 | 44.27916667 |
|  |  |  |  |  |  | 77.4875 | 37.70416667 |  |  |  |  | 82.82083333 | 45.0875 |
|  |  |  |  |  |  | 77.52916667 | 37.42083333 |  |  |  |  | 82.82916667 | 44.45416667 |
|  |  |  |  |  |  | 77.54583333 | 37.37916667 |  |  |  |  | 82.8625 | 44.5875 |
|  |  |  |  |  |  | 77.55416667 | 39.57083333 |  |  |  |  | 82.89583333 | 44.59583333 |
|  |  |  |  |  |  | 77.5875 | 39.64583333 |  |  |  |  | 82.89583333 | 45.05416667 |
|  |  |  |  |  |  | 77.70416667 | 38.7875 |  |  |  |  | 82.92916667 | 44.42916667 |
|  |  |  |  |  |  | 77.7125 | 38.7125 |  |  |  |  | 82.94583333 | 44.44583333 |
|  |  |  |  |  |  | 77.7375 | 38.8625 |  |  |  |  | 82.94583333 | 44.4625 |
|  |  |  |  |  |  | 77.7875 | 38.6625 |  |  |  |  | 82.95416667 | 44.5125 |
|  |  |  |  |  |  | 77.82083333 | 38.62916667 |  |  |  |  | 82.9625 | 44.45416667 |
|  |  |  |  |  |  | 77.82916667 | 38.60416667 |  |  |  |  | 82.9625 | 44.5625 |
|  |  |  |  |  |  | 77.82916667 | 38.62916667 |  |  |  |  | 82.97083333 | 45.02083333 |
|  |  |  |  |  |  | 77.94583333 | 38.42916667 |  |  |  |  | 82.97916667 | 44.4625 |
|  |  |  |  |  |  | 77.99583333 | 46.00416667 |  |  |  |  | 82.9875 | 44.47083333 |
|  |  |  |  |  |  | 78.27083333 | 37.0125 |  |  |  |  | 83.00416667 | 44.45416667 |
|  |  |  |  |  |  | 78.27916667 | 39.7125 |  |  |  |  | 83.0125 | 44.44583333 |
|  |  |  |  |  |  | 78.2875 | 37.95416667 |  |  |  |  | 83.0125 | 44.4875 |
|  |  |  |  |  |  | 78.35416667 | 37.5125 |  |  |  |  | 83.0125 | 44.49583333 |
|  |  |  |  |  |  | 78.3875 | 37.85416667 |  |  |  |  | 83.02916667 | 44.40416667 |
|  |  |  |  |  |  | 78.44583333 | 37.8125 |  |  |  |  | 83.02916667 | 44.42083333 |
|  |  |  |  |  |  | 78.4625 | 37.6625 |  |  |  |  | 83.0375 | 44.44583333 |
|  |  |  |  |  |  | 78.4625 | 37.67916667 |  |  |  |  | 83.0375 | 44.49583333 |
|  |  |  |  |  |  | 78.49583333 | 37.7875 |  |  |  |  | 83.04583333 | 44.47916667 |
|  |  |  |  |  |  | 78.5125 | 37.74583333 |  |  |  |  | 83.04583333 | 44.62083333 |
|  |  |  |  |  |  | 78.52083333 | 37.74583333 |  |  |  |  | 83.07083333 | 44.42916667 |
|  |  |  |  |  |  | 78.5875 | 40.5375 |  |  |  |  | 83.07916667 | 44.4625 |
|  |  |  |  |  |  | 78.59583333 | 40.52916667 |  |  |  |  | 83.09583333 | 44.45416667 |
|  |  |  |  |  |  | 78.67083333 | 39.65416667 |  |  |  |  | 83.10416667 | 44.6125 |
|  |  |  |  |  |  | 78.72916667 | 39.90416667 |  |  |  |  | 83.14583333 | 44.62083333 |
|  |  |  |  |  |  | 78.7875 | 41.17083333 |  |  |  |  | 83.17916667 | 44.5375 |
|  |  |  |  |  |  | 78.8125 | 41.10416667 |  |  |  |  | 83.1875 | 45.0125 |
|  |  |  |  |  |  | 78.94583333 | 41.19583333 |  |  |  |  | 83.47916667 | 45.02083333 |
|  |  |  |  |  |  | 79.15416667 | 40.4875 |  |  |  |  | 83.57916667 | 45.0125 |
|  |  |  |  |  |  | 79.25416667 | 40.49583333 |  |  |  |  | 83.72916667 | 44.95416667 |
|  |  |  |  |  |  | 79.3375 | 40.3625 |  |  |  |  | 83.74583333 | 44.9625 |
|  |  |  |  |  |  | 79.39583333 | 40.20416667 |  |  |  |  | 83.87083333 | 44.72083333 |
|  |  |  |  |  |  | 79.4125 | 36.92916667 |  |  |  |  | 83.89583333 | 40.8375 |
|  |  |  |  |  |  | 79.42083333 | 36.87916667 |  |  |  |  | 83.89583333 | 45.0375 |
|  |  |  |  |  |  | 79.42916667 | 36.87916667 |  |  |  |  | 83.92083333 | 45.17083333 |
|  |  |  |  |  |  | 79.47916667 | 36.90416667 |  |  |  |  | 83.9375 | 45.12916667 |
|  |  |  |  |  |  | 79.49583333 | 37.1375 |  |  |  |  | 84.47083333 | 46.1375 |
|  |  |  |  |  |  | 79.50416667 | 37.24583333 |  |  |  |  | 84.52083333 | 46.1375 |
|  |  |  |  |  |  | 79.60416667 | 37.5625 |  |  |  |  | 84.6625 | 45.22916667 |
|  |  |  |  |  |  | 79.6125 | 37.5625 |  |  |  |  | 84.67083333 | 45.24583333 |
|  |  |  |  |  |  | 79.62916667 | 40.55416667 |  |  |  |  | 84.6875 | 45.20416667 |
|  |  |  |  |  |  | 79.6625 | 36.92083333 |  |  |  |  | 84.6875 | 45.2625 |
|  |  |  |  |  |  | 79.70416667 | 36.94583333 |  |  |  |  | 84.69583333 | 45.12916667 |
|  |  |  |  |  |  | 79.70416667 | 36.95416667 |  |  |  |  | 84.69583333 | 45.1625 |
|  |  |  |  |  |  | 79.85416667 | 37.2625 |  |  |  |  | 84.69583333 | 45.17083333 |
|  |  |  |  |  |  | 79.8625 | 41.10416667 |  |  |  |  | 84.69583333 | 45.17916667 |
|  |  |  |  |  |  | 79.87083333 | 36.64583333 |  |  |  |  | 84.70416667 | 45.27083333 |
|  |  |  |  |  |  | 79.9125 | 37.35416667 |  |  |  |  | 84.72083333 | 45.29583333 |
|  |  |  |  |  |  | 79.94583333 | 36.34583333 |  |  |  |  | 84.7375 | 45.3125 |
|  |  |  |  |  |  | 79.94583333 | 37.42083333 |  |  |  |  | 84.74583333 | 45.09583333 |
|  |  |  |  |  |  | 79.94583333 | 41.10416667 |  |  |  |  | 84.75416667 | 45.10416667 |
|  |  |  |  |  |  | 79.99583333 | 37.30416667 |  |  |  |  | 84.75416667 | 45.3375 |
|  |  |  |  |  |  | 80.1375 | 37.55416667 |  |  |  |  | 84.77083333 | 45.3625 |
|  |  |  |  |  |  | 80.14583333 | 37.5625 |  |  |  |  | 84.80416667 | 45.07916667 |
|  |  |  |  |  |  | 80.19583333 | 41.10416667 |  |  |  |  | 84.84583333 | 45.09583333 |
|  |  |  |  |  |  | 80.2125 | 37.3125 |  |  |  |  | 84.87083333 | 45.0625 |
|  |  |  |  |  |  | 80.22916667 | 37.72916667 |  |  |  |  | 84.87916667 | 45.09583333 |
|  |  |  |  |  |  | 80.2625 | 37.62916667 |  |  |  |  | 84.8875 | 45.0625 |
|  |  |  |  |  |  | 80.27083333 | 37.62916667 |  |  |  |  | 84.89583333 | 45.04583333 |
|  |  |  |  |  |  | 80.27916667 | 37.67916667 |  |  |  |  | 84.89583333 | 45.50416667 |
|  |  |  |  |  |  | 80.2875 | 37.19583333 |  |  |  |  | 84.90416667 | 45.00416667 |
|  |  |  |  |  |  | 80.35416667 | 37.7875 |  |  |  |  | 84.9125 | 44.84583333 |
|  |  |  |  |  |  | 80.3625 | 37.27083333 |  |  |  |  | 84.9125 | 44.9875 |
|  |  |  |  |  |  | 80.3625 | 37.7875 |  |  |  |  | 84.9125 | 45.04583333 |
|  |  |  |  |  |  | 80.3625 | 41.72916667 |  |  |  |  | 84.92083333 | 44.90416667 |
|  |  |  |  |  |  | 80.39583333 | 44.20416667 |  |  |  |  | 84.92083333 | 44.92083333 |
|  |  |  |  |  |  | 80.4125 | 37.1125 |  |  |  |  | 84.92916667 | 44.82916667 |
|  |  |  |  |  |  | 80.4125 | 37.17916667 |  |  |  |  | 84.92916667 | 44.8875 |
|  |  |  |  |  |  | 80.42083333 | 37.82916667 |  |  |  |  | 84.97083333 | 44.84583333 |
|  |  |  |  |  |  | 80.5875 | 36.3875 |  |  |  |  | 84.99583333 | 44.80416667 |
|  |  |  |  |  |  | 80.59583333 | 37.0625 |  |  |  |  | 85.04583333 | 44.34583333 |
|  |  |  |  |  |  | 80.60416667 | 41.7375 |  |  |  |  | 85.04583333 | 44.3625 |
|  |  |  |  |  |  | 80.64583333 | 37.02916667 |  |  |  |  | 85.19583333 | 45.75416667 |
|  |  |  |  |  |  | 80.65416667 | 40.35416667 |  |  |  |  | 85.2125 | 45.7625 |
|  |  |  |  |  |  | 80.65416667 | 40.89583333 |  |  |  |  | 85.22083333 | 45.77083333 |
|  |  |  |  |  |  | 80.65416667 | 40.92083333 |  |  |  |  | 85.2375 | 45.7875 |
|  |  |  |  |  |  | 80.67083333 | 40.92083333 |  |  |  |  | 85.25416667 | 45.82916667 |
|  |  |  |  |  |  | 80.67916667 | 41.45416667 |  |  |  |  | 85.27083333 | 45.8875 |
|  |  |  |  |  |  | 80.69583333 | 37.0125 |  |  |  |  | 85.2875 | 45.92083333 |
|  |  |  |  |  |  | 80.72083333 | 36.4375 |  |  |  |  | 85.32916667 | 45.97916667 |
|  |  |  |  |  |  | 80.7375 | 36.99583333 |  |  |  |  | 85.3375 | 46.4875 |
|  |  |  |  |  |  | 80.74583333 | 36.5625 |  |  |  |  | 85.34583333 | 46.02083333 |
|  |  |  |  |  |  | 80.74583333 | 43.9375 |  |  |  |  | 85.34583333 | 46.04583333 |
|  |  |  |  |  |  | 80.74583333 | 43.94583333 |  |  |  |  | 85.3625 | 45.75416667 |
|  |  |  |  |  |  | 80.75416667 | 43.92916667 |  |  |  |  | 85.37083333 | 46.49583333 |
|  |  |  |  |  |  | 80.75416667 | 43.99583333 |  |  |  |  | 85.37916667 | 46.05416667 |
|  |  |  |  |  |  | 80.7625 | 44.02083333 |  |  |  |  | 85.37916667 | 46.0625 |
|  |  |  |  |  |  | 80.77916667 | 41.47916667 |  |  |  |  | 85.39583333 | 45.75416667 |
|  |  |  |  |  |  | 80.77916667 | 43.92916667 |  |  |  |  | 85.39583333 | 45.77083333 |
|  |  |  |  |  |  | 80.77916667 | 43.97916667 |  |  |  |  | 85.40416667 | 45.90416667 |
|  |  |  |  |  |  | 80.79583333 | 41.15416667 |  |  |  |  | 85.45416667 | 46.50416667 |
|  |  |  |  |  |  | 80.80416667 | 36.94583333 |  |  |  |  | 85.49583333 | 46.5625 |
|  |  |  |  |  |  | 80.80416667 | 43.9375 |  |  |  |  | 85.50416667 | 45.50416667 |
|  |  |  |  |  |  | 80.82083333 | 36.2625 |  |  |  |  | 85.52916667 | 46.5625 |
|  |  |  |  |  |  | 80.82083333 | 41.14583333 |  |  |  |  | 85.55416667 | 45.49583333 |
|  |  |  |  |  |  | 80.94583333 | 36.8875 |  |  |  |  | 85.5875 | 45.49583333 |
|  |  |  |  |  |  | 80.94583333 | 44.0375 |  |  |  |  | 85.67083333 | 45.47916667 |
|  |  |  |  |  |  | 80.95416667 | 44.22916667 |  |  |  |  | 85.67083333 | 46.57083333 |
|  |  |  |  |  |  | 80.9875 | 44.32083333 |  |  |  |  | 85.7125 | 46.8125 |
|  |  |  |  |  |  | 81.0125 | 36.37916667 |  |  |  |  | 85.72916667 | 46.5625 |
|  |  |  |  |  |  | 81.02083333 | 36.4625 |  |  |  |  | 85.75416667 | 46.69583333 |
|  |  |  |  |  |  | 81.02916667 | 36.79583333 |  |  |  |  | 85.75416667 | 46.7375 |
|  |  |  |  |  |  | 81.0375 | 36.7875 |  |  |  |  | 85.75416667 | 46.74583333 |
|  |  |  |  |  |  | 81.04583333 | 44.1125 |  |  |  |  | 85.7625 | 46.82916667 |
|  |  |  |  |  |  | 81.07083333 | 44.12916667 |  |  |  |  | 85.7875 | 46.55416667 |
|  |  |  |  |  |  | 81.07916667 | 44.1625 |  |  |  |  | 85.79583333 | 45.47916667 |
|  |  |  |  |  |  | 81.12916667 | 36.32083333 |  |  |  |  | 85.8125 | 46.1875 |
|  |  |  |  |  |  | 81.12916667 | 45.0125 |  |  |  |  | 85.82916667 | 46.82083333 |
|  |  |  |  |  |  | 81.15416667 | 36.97083333 |  |  |  |  | 85.8375 | 46.84583333 |
|  |  |  |  |  |  | 81.1625 | 36.97083333 |  |  |  |  | 85.8375 | 46.85416667 |
|  |  |  |  |  |  | 81.1625 | 40.89583333 |  |  |  |  | 85.84583333 | 46.22916667 |
|  |  |  |  |  |  | 81.17916667 | 36.9625 |  |  |  |  | 85.85416667 | 45.47916667 |
|  |  |  |  |  |  | 81.19583333 | 36.3375 |  |  |  |  | 85.87083333 | 46.2375 |
|  |  |  |  |  |  | 81.19583333 | 41.77916667 |  |  |  |  | 85.87083333 | 46.52916667 |
|  |  |  |  |  |  | 81.22083333 | 36.9375 |  |  |  |  | 85.8875 | 46.6125 |
|  |  |  |  |  |  | 81.30416667 | 41.7375 |  |  |  |  | 85.89583333 | 46.25416667 |
|  |  |  |  |  |  | 81.30416667 | 41.8875 |  |  |  |  | 85.90416667 | 46.92083333 |
|  |  |  |  |  |  | 81.3625 | 37.4625 |  |  |  |  | 85.9125 | 46.29583333 |
|  |  |  |  |  |  | 81.37916667 | 41.87916667 |  |  |  |  | 85.92083333 | 46.32083333 |
|  |  |  |  |  |  | 81.3875 | 41.72916667 |  |  |  |  | 85.92916667 | 46.32083333 |
|  |  |  |  |  |  | 81.42916667 | 41.5875 |  |  |  |  | 85.92916667 | 46.5375 |
|  |  |  |  |  |  | 81.4375 | 41.6875 |  |  |  |  | 85.9375 | 46.3625 |
|  |  |  |  |  |  | 81.47916667 | 37.2375 |  |  |  |  | 85.9375 | 46.9625 |
|  |  |  |  |  |  | 81.5125 | 37.0125 |  |  |  |  | 85.94583333 | 45.47083333 |
|  |  |  |  |  |  | 81.5375 | 37.10416667 |  |  |  |  | 85.95416667 | 46.4125 |
|  |  |  |  |  |  | 81.54583333 | 41.62083333 |  |  |  |  | 85.9625 | 46.42916667 |
|  |  |  |  |  |  | 81.57083333 | 41.7875 |  |  |  |  | 85.9625 | 46.82083333 |
|  |  |  |  |  |  | 81.57916667 | 41.40416667 |  |  |  |  | 85.9625 | 47.69583333 |
|  |  |  |  |  |  | 81.62083333 | 41.85416667 |  |  |  |  | 85.97083333 | 44.72916667 |
|  |  |  |  |  |  | 81.64583333 | 41.62083333 |  |  |  |  | 85.97083333 | 46.20416667 |
|  |  |  |  |  |  | 81.82083333 | 41.8625 |  |  |  |  | 85.97083333 | 46.5875 |
|  |  |  |  |  |  | 81.8875 | 41.20416667 |  |  |  |  | 85.9875 | 46.52083333 |
|  |  |  |  |  |  | 81.89583333 | 36.62916667 |  |  |  |  | 86.00416667 | 46.82916667 |
|  |  |  |  |  |  | 81.9375 | 36.5125 |  |  |  |  | 86.02083333 | 46.4875 |
|  |  |  |  |  |  | 82.0125 | 44.45416667 |  |  |  |  | 86.02083333 | 46.5875 |
|  |  |  |  |  |  | 82.02083333 | 44.6375 |  |  |  |  | 86.02916667 | 44.0125 |
|  |  |  |  |  |  | 82.09583333 | 36.74583333 |  |  |  |  | 86.02916667 | 45.47083333 |
|  |  |  |  |  |  | 82.12083333 | 41.97083333 |  |  |  |  | 86.0375 | 46.7625 |
|  |  |  |  |  |  | 82.14583333 | 41.3125 |  |  |  |  | 86.04583333 | 46.82083333 |
|  |  |  |  |  |  | 82.15416667 | 40.84583333 |  |  |  |  | 86.0625 | 44.75416667 |
|  |  |  |  |  |  | 82.19583333 | 46.40416667 |  |  |  |  | 86.07083333 | 46.67083333 |
|  |  |  |  |  |  | 82.2625 | 41.39583333 |  |  |  |  | 86.07916667 | 44.79583333 |
|  |  |  |  |  |  | 82.2625 | 43.24583333 |  |  |  |  | 86.0875 | 46.7375 |
|  |  |  |  |  |  | 82.29583333 | 45.00416667 |  |  |  |  | 86.09583333 | 44.60416667 |
|  |  |  |  |  |  | 82.29583333 | 45.10416667 |  |  |  |  | 86.10416667 | 46.8375 |
|  |  |  |  |  |  | 82.30416667 | 43.2625 |  |  |  |  | 86.12083333 | 45.4625 |
|  |  |  |  |  |  | 82.3625 | 42.1625 |  |  |  |  | 86.12916667 | 46.72916667 |
|  |  |  |  |  |  | 82.37083333 | 44.3375 |  |  |  |  | 86.1375 | 44.77916667 |
|  |  |  |  |  |  | 82.37916667 | 42.15416667 |  |  |  |  | 86.1375 | 47.77916667 |
|  |  |  |  |  |  | 82.42083333 | 42.07083333 |  |  |  |  | 86.14583333 | 46.12083333 |
|  |  |  |  |  |  | 82.42083333 | 43.39583333 |  |  |  |  | 86.14583333 | 46.72916667 |
|  |  |  |  |  |  | 82.44583333 | 42.14583333 |  |  |  |  | 86.17083333 | 46.7375 |
|  |  |  |  |  |  | 82.45416667 | 44.9375 |  |  |  |  | 86.17916667 | 46.1125 |
|  |  |  |  |  |  | 82.4625 | 42.07916667 |  |  |  |  | 86.17916667 | 46.8625 |
|  |  |  |  |  |  | 82.47916667 | 44.90416667 |  |  |  |  | 86.19583333 | 44.74583333 |
|  |  |  |  |  |  | 82.49583333 | 45.00416667 |  |  |  |  | 86.20416667 | 46.14583333 |
|  |  |  |  |  |  | 82.5625 | 41.17916667 |  |  |  |  | 86.2125 | 45.44583333 |
|  |  |  |  |  |  | 82.6625 | 36.6125 |  |  |  |  | 86.22916667 | 46.8625 |
|  |  |  |  |  |  | 82.67916667 | 36.97916667 |  |  |  |  | 86.2375 | 44.70416667 |
|  |  |  |  |  |  | 82.67916667 | 40.95416667 |  |  |  |  | 86.24583333 | 46.17083333 |
|  |  |  |  |  |  | 82.69583333 | 37.02916667 |  |  |  |  | 86.24583333 | 46.2625 |
|  |  |  |  |  |  | 82.77083333 | 37.59583333 |  |  |  |  | 86.25416667 | 46.7875 |
|  |  |  |  |  |  | 82.77083333 | 44.34583333 |  |  |  |  | 86.25416667 | 46.87083333 |
|  |  |  |  |  |  | 82.77916667 | 44.37916667 |  |  |  |  | 86.2625 | 46.7875 |
|  |  |  |  |  |  | 82.79583333 | 37.19583333 |  |  |  |  | 86.27083333 | 46.1875 |
|  |  |  |  |  |  | 82.79583333 | 37.20416667 |  |  |  |  | 86.2875 | 44.67916667 |
|  |  |  |  |  |  | 82.79583333 | 37.72083333 |  |  |  |  | 86.2875 | 46.07083333 |
|  |  |  |  |  |  | 82.79583333 | 44.27916667 |  |  |  |  | 86.29583333 | 44.7375 |
|  |  |  |  |  |  | 82.8125 | 37.34583333 |  |  |  |  | 86.30416667 | 45.39583333 |
|  |  |  |  |  |  | 82.82916667 | 44.45416667 |  |  |  |  | 86.30416667 | 45.4125 |
|  |  |  |  |  |  | 82.85416667 | 37.42916667 |  |  |  |  | 86.30416667 | 45.45416667 |
|  |  |  |  |  |  | 82.8625 | 37.47916667 |  |  |  |  | 86.3125 | 45.4875 |
|  |  |  |  |  |  | 82.87916667 | 37.5625 |  |  |  |  | 86.3125 | 46.85416667 |
|  |  |  |  |  |  | 82.8875 | 37.62916667 |  |  |  |  | 86.32916667 | 44.75416667 |
|  |  |  |  |  |  | 82.9375 | 37.22083333 |  |  |  |  | 86.32916667 | 46.2375 |
|  |  |  |  |  |  | 82.94583333 | 37.69583333 |  |  |  |  | 86.3375 | 44.0375 |
|  |  |  |  |  |  | 82.95416667 | 37.04583333 |  |  |  |  | 86.3375 | 44.6625 |
|  |  |  |  |  |  | 82.97083333 | 45.02083333 |  |  |  |  | 86.3375 | 45.52083333 |
|  |  |  |  |  |  | 82.97916667 | 41.95416667 |  |  |  |  | 86.34583333 | 44.64583333 |
|  |  |  |  |  |  | 82.97916667 | 44.50416667 |  |  |  |  | 86.34583333 | 44.6625 |
|  |  |  |  |  |  | 83.00416667 | 37.25416667 |  |  |  |  | 86.35416667 | 45.57916667 |
|  |  |  |  |  |  | 83.0125 | 37.74583333 |  |  |  |  | 86.3625 | 45.44583333 |
|  |  |  |  |  |  | 83.02916667 | 37.90416667 |  |  |  |  | 86.37916667 | 46.02083333 |
|  |  |  |  |  |  | 83.0375 | 44.62083333 |  |  |  |  | 86.3875 | 45.64583333 |
|  |  |  |  |  |  | 83.0375 | 44.62916667 |  |  |  |  | 86.40416667 | 47.1875 |
|  |  |  |  |  |  | 83.04583333 | 44.62083333 |  |  |  |  | 86.4125 | 44.67916667 |
|  |  |  |  |  |  | 83.0625 | 44.6125 |  |  |  |  | 86.4125 | 47.2125 |
|  |  |  |  |  |  | 83.07916667 | 37.27916667 |  |  |  |  | 86.42083333 | 45.67916667 |
|  |  |  |  |  |  | 83.12916667 | 37.9125 |  |  |  |  | 86.42083333 | 45.8125 |
|  |  |  |  |  |  | 83.14583333 | 41.27083333 |  |  |  |  | 86.42916667 | 45.44583333 |
|  |  |  |  |  |  | 83.1625 | 36.72083333 |  |  |  |  | 86.42916667 | 45.8125 |
|  |  |  |  |  |  | 83.19583333 | 37.3375 |  |  |  |  | 86.42916667 | 46.89583333 |
|  |  |  |  |  |  | 83.2125 | 44.59583333 |  |  |  |  | 86.42916667 | 46.9125 |
|  |  |  |  |  |  | 83.22083333 | 45.00416667 |  |  |  |  | 86.4375 | 45.70416667 |
|  |  |  |  |  |  | 83.2375 | 37.9125 |  |  |  |  | 86.4375 | 45.77083333 |
|  |  |  |  |  |  | 83.2625 | 37.3625 |  |  |  |  | 86.4375 | 47.17916667 |
|  |  |  |  |  |  | 83.2875 | 41.62083333 |  |  |  |  | 86.45416667 | 46.0625 |
|  |  |  |  |  |  | 83.3375 | 44.99583333 |  |  |  |  | 86.47916667 | 45.87083333 |
|  |  |  |  |  |  | 83.37916667 | 41.62083333 |  |  |  |  | 86.47916667 | 46.09583333 |
|  |  |  |  |  |  | 83.37916667 | 41.6375 |  |  |  |  | 86.4875 | 46.0125 |
|  |  |  |  |  |  | 83.3875 | 36.9375 |  |  |  |  | 86.49583333 | 46.9375 |
|  |  |  |  |  |  | 83.39583333 | 39.00416667 |  |  |  |  | 86.50416667 | 44.90416667 |
|  |  |  |  |  |  | 83.42083333 | 41.77083333 |  |  |  |  | 86.5125 | 46.07916667 |
|  |  |  |  |  |  | 83.42916667 | 36.8625 |  |  |  |  | 86.5125 | 46.82916667 |
|  |  |  |  |  |  | 83.47916667 | 36.82916667 |  |  |  |  | 86.52916667 | 44.92083333 |
|  |  |  |  |  |  | 83.47916667 | 41.5625 |  |  |  |  | 86.52916667 | 45.44583333 |
|  |  |  |  |  |  | 83.5125 | 41.72083333 |  |  |  |  | 86.52916667 | 45.92916667 |
|  |  |  |  |  |  | 83.5125 | 41.77083333 |  |  |  |  | 86.52916667 | 46.02083333 |
|  |  |  |  |  |  | 83.52083333 | 36.77916667 |  |  |  |  | 86.52916667 | 46.82083333 |
|  |  |  |  |  |  | 83.52916667 | 41.2875 |  |  |  |  | 86.5375 | 45.92916667 |
|  |  |  |  |  |  | 83.54583333 | 41.45416667 |  |  |  |  | 86.54583333 | 46.82083333 |
|  |  |  |  |  |  | 83.54583333 | 41.54583333 |  |  |  |  | 86.55416667 | 46.72916667 |
|  |  |  |  |  |  | 83.55416667 | 37.4625 |  |  |  |  | 86.5625 | 46.72083333 |
|  |  |  |  |  |  | 83.5625 | 41.60416667 |  |  |  |  | 86.57083333 | 46.9625 |
|  |  |  |  |  |  | 83.57083333 | 41.42083333 |  |  |  |  | 86.59583333 | 46.6875 |
|  |  |  |  |  |  | 83.57916667 | 37.94583333 |  |  |  |  | 86.6125 | 44.9125 |
|  |  |  |  |  |  | 83.60416667 | 36.72916667 |  |  |  |  | 86.62083333 | 45.4625 |
|  |  |  |  |  |  | 83.64583333 | 37.95416667 |  |  |  |  | 86.62083333 | 46.52083333 |
|  |  |  |  |  |  | 83.6875 | 37.87083333 |  |  |  |  | 86.64583333 | 44.84583333 |
|  |  |  |  |  |  | 83.70416667 | 41.12083333 |  |  |  |  | 86.64583333 | 46.97916667 |
|  |  |  |  |  |  | 83.72083333 | 37.79583333 |  |  |  |  | 86.65416667 | 46.7375 |
|  |  |  |  |  |  | 83.72916667 | 37.72083333 |  |  |  |  | 86.6625 | 47.17083333 |
|  |  |  |  |  |  | 83.72916667 | 41.4125 |  |  |  |  | 86.67083333 | 44.8125 |
|  |  |  |  |  |  | 83.7375 | 37.5375 |  |  |  |  | 86.67083333 | 45.4625 |
|  |  |  |  |  |  | 83.77083333 | 37.5625 |  |  |  |  | 86.67916667 | 46.7125 |
|  |  |  |  |  |  | 83.77083333 | 41.37083333 |  |  |  |  | 86.6875 | 44.77916667 |
|  |  |  |  |  |  | 83.77083333 | 41.3875 |  |  |  |  | 86.70416667 | 44.7625 |
|  |  |  |  |  |  | 83.77916667 | 41.32916667 |  |  |  |  | 86.7125 | 44.79583333 |
|  |  |  |  |  |  | 83.79583333 | 41.3875 |  |  |  |  | 86.7375 | 45.45416667 |
|  |  |  |  |  |  | 83.80416667 | 37.6375 |  |  |  |  | 86.74583333 | 45.89583333 |
|  |  |  |  |  |  | 83.82083333 | 41.30416667 |  |  |  |  | 86.75416667 | 46.4625 |
|  |  |  |  |  |  | 83.82916667 | 36.82083333 |  |  |  |  | 86.75416667 | 46.99583333 |
|  |  |  |  |  |  | 83.8375 | 36.8625 |  |  |  |  | 86.7625 | 46.39583333 |
|  |  |  |  |  |  | 83.84583333 | 41.07083333 |  |  |  |  | 86.77916667 | 45.92083333 |
|  |  |  |  |  |  | 83.90416667 | 41.24583333 |  |  |  |  | 86.7875 | 45.60416667 |
|  |  |  |  |  |  | 83.90416667 | 41.27083333 |  |  |  |  | 86.79583333 | 44.7625 |
|  |  |  |  |  |  | 83.90416667 | 41.37916667 |  |  |  |  | 86.79583333 | 45.62916667 |
|  |  |  |  |  |  | 83.9125 | 41.27083333 |  |  |  |  | 86.79583333 | 46.27083333 |
|  |  |  |  |  |  | 83.92916667 | 41.4125 |  |  |  |  | 86.82916667 | 44.77916667 |
|  |  |  |  |  |  | 83.9375 | 41.09583333 |  |  |  |  | 86.82916667 | 45.44583333 |
|  |  |  |  |  |  | 83.9375 | 41.42916667 |  |  |  |  | 86.84583333 | 45.4375 |
|  |  |  |  |  |  | 83.94583333 | 41.34583333 |  |  |  |  | 86.84583333 | 45.75416667 |
|  |  |  |  |  |  | 83.94583333 | 41.50416667 |  |  |  |  | 86.8625 | 45.52083333 |
|  |  |  |  |  |  | 83.95416667 | 41.3375 |  |  |  |  | 86.87083333 | 45.47916667 |
|  |  |  |  |  |  | 83.97083333 | 41.3375 |  |  |  |  | 86.87083333 | 45.9875 |
|  |  |  |  |  |  | 83.97916667 | 40.92916667 |  |  |  |  | 86.87916667 | 44.6625 |
|  |  |  |  |  |  | 83.97916667 | 41.3125 |  |  |  |  | 86.87916667 | 44.77083333 |
|  |  |  |  |  |  | 84.00416667 | 41.32916667 |  |  |  |  | 86.87916667 | 45.4125 |
|  |  |  |  |  |  | 84.00416667 | 41.40416667 |  |  |  |  | 86.87916667 | 47.54583333 |
|  |  |  |  |  |  | 84.17916667 | 37.6875 |  |  |  |  | 86.8875 | 45.84583333 |
|  |  |  |  |  |  | 84.1875 | 41.74583333 |  |  |  |  | 86.89583333 | 45.85416667 |
|  |  |  |  |  |  | 84.22083333 | 41.65416667 |  |  |  |  | 86.90416667 | 46.02916667 |
|  |  |  |  |  |  | 84.2625 | 41.57083333 |  |  |  |  | 86.9125 | 46.37083333 |
|  |  |  |  |  |  | 84.27916667 | 41.55416667 |  |  |  |  | 86.92083333 | 46.05416667 |
|  |  |  |  |  |  | 84.27916667 | 41.5625 |  |  |  |  | 86.92083333 | 47.00416667 |
|  |  |  |  |  |  | 84.37916667 | 41.47916667 |  |  |  |  | 86.92916667 | 46.2625 |
|  |  |  |  |  |  | 84.3875 | 41.4875 |  |  |  |  | 86.9375 | 44.45416667 |
|  |  |  |  |  |  | 84.4375 | 41.27916667 |  |  |  |  | 86.9375 | 44.75416667 |
|  |  |  |  |  |  | 84.47083333 | 44.15416667 |  |  |  |  | 86.94583333 | 44.57083333 |
|  |  |  |  |  |  | 84.50416667 | 41.2625 |  |  |  |  | 86.95416667 | 46.10416667 |
|  |  |  |  |  |  | 84.60416667 | 38.00416667 |  |  |  |  | 86.9625 | 44.57916667 |
|  |  |  |  |  |  | 84.65416667 | 38.02083333 |  |  |  |  | 86.97083333 | 44.74583333 |
|  |  |  |  |  |  | 84.65416667 | 45.3375 |  |  |  |  | 86.97083333 | 46.1375 |
|  |  |  |  |  |  | 84.67916667 | 41.89583333 |  |  |  |  | 86.9875 | 45.45416667 |
|  |  |  |  |  |  | 84.67916667 | 45.10416667 |  |  |  |  | 86.99583333 | 47.0125 |
|  |  |  |  |  |  | 84.69583333 | 45.32916667 |  |  |  |  | 87.0125 | 44.7375 |
|  |  |  |  |  |  | 84.70416667 | 45.2875 |  |  |  |  | 87.02083333 | 46.2125 |
|  |  |  |  |  |  | 84.77083333 | 41.97083333 |  |  |  |  | 87.02916667 | 44.67916667 |
|  |  |  |  |  |  | 84.90416667 | 38.14583333 |  |  |  |  | 87.02916667 | 44.70416667 |
|  |  |  |  |  |  | 84.92083333 | 44.9125 |  |  |  |  | 87.02916667 | 45.42916667 |
|  |  |  |  |  |  | 84.9375 | 44.8875 |  |  |  |  | 87.02916667 | 46.22916667 |
|  |  |  |  |  |  | 84.94583333 | 38.1875 |  |  |  |  | 87.0375 | 44.74583333 |
|  |  |  |  |  |  | 84.94583333 | 44.8375 |  |  |  |  | 87.0375 | 44.75416667 |
|  |  |  |  |  |  | 84.95416667 | 44.87083333 |  |  |  |  | 87.04583333 | 44.55416667 |
|  |  |  |  |  |  | 84.99583333 | 37.20416667 |  |  |  |  | 87.04583333 | 45.40416667 |
|  |  |  |  |  |  | 85.09583333 | 47.90416667 |  |  |  |  | 87.05416667 | 44.62083333 |
|  |  |  |  |  |  | 85.1375 | 45.72916667 |  |  |  |  | 87.0625 | 44.57083333 |
|  |  |  |  |  |  | 85.2375 | 45.55416667 |  |  |  |  | 87.0625 | 44.67083333 |
|  |  |  |  |  |  | 85.24583333 | 45.79583333 |  |  |  |  | 87.0875 | 44.59583333 |
|  |  |  |  |  |  | 85.2625 | 45.5625 |  |  |  |  | 87.0875 | 44.6125 |
|  |  |  |  |  |  | 85.29583333 | 38.2375 |  |  |  |  | 87.0875 | 44.65416667 |
|  |  |  |  |  |  | 85.3125 | 45.94583333 |  |  |  |  | 87.1375 | 44.64583333 |
|  |  |  |  |  |  | 85.3125 | 46.00416667 |  |  |  |  | 87.14583333 | 45.32916667 |
|  |  |  |  |  |  | 85.3125 | 46.02083333 |  |  |  |  | 87.15416667 | 45.37083333 |
|  |  |  |  |  |  | 85.34583333 | 46.02083333 |  |  |  |  | 87.19583333 | 44.6375 |
|  |  |  |  |  |  | 85.37916667 | 46.05416667 |  |  |  |  | 87.2375 | 44.47916667 |
|  |  |  |  |  |  | 85.37916667 | 46.0625 |  |  |  |  | 87.2375 | 44.69583333 |
|  |  |  |  |  |  | 85.45416667 | 38.2125 |  |  |  |  | 87.24583333 | 44.4375 |
|  |  |  |  |  |  | 85.49583333 | 37.30416667 |  |  |  |  | 87.25416667 | 44.3875 |
|  |  |  |  |  |  | 85.49583333 | 38.30416667 |  |  |  |  | 87.25416667 | 44.69583333 |
|  |  |  |  |  |  | 85.49583333 | 47.00416667 |  |  |  |  | 87.2625 | 44.3125 |
|  |  |  |  |  |  | 85.69583333 | 46.79583333 |  |  |  |  | 87.2625 | 44.37083333 |
|  |  |  |  |  |  | 85.7625 | 38.50416667 |  |  |  |  | 87.2625 | 46.32083333 |
|  |  |  |  |  |  | 85.77083333 | 37.6125 |  |  |  |  | 87.27083333 | 44.55416667 |
|  |  |  |  |  |  | 85.77916667 | 44.17916667 |  |  |  |  | 87.27916667 | 44.3875 |
|  |  |  |  |  |  | 85.79583333 | 38.47083333 |  |  |  |  | 87.2875 | 46.8125 |
|  |  |  |  |  |  | 85.79583333 | 44.12916667 |  |  |  |  | 87.29583333 | 44.82083333 |
|  |  |  |  |  |  | 85.80416667 | 37.57083333 |  |  |  |  | 87.29583333 | 44.82916667 |
|  |  |  |  |  |  | 85.82083333 | 41.7125 |  |  |  |  | 87.29583333 | 44.87916667 |
|  |  |  |  |  |  | 85.82083333 | 44.2375 |  |  |  |  | 87.29583333 | 44.8875 |
|  |  |  |  |  |  | 85.87083333 | 42.22083333 |  |  |  |  | 87.29583333 | 44.9125 |
|  |  |  |  |  |  | 85.87916667 | 41.47083333 |  |  |  |  | 87.29583333 | 44.9375 |
|  |  |  |  |  |  | 85.89583333 | 37.50416667 |  |  |  |  | 87.30416667 | 44.79583333 |
|  |  |  |  |  |  | 85.90416667 | 41.14583333 |  |  |  |  | 87.30416667 | 46.85416667 |
|  |  |  |  |  |  | 85.9125 | 47.69583333 |  |  |  |  | 87.3125 | 44.7625 |
|  |  |  |  |  |  | 85.9375 | 46.8125 |  |  |  |  | 87.3125 | 44.77083333 |
|  |  |  |  |  |  | 85.97083333 | 37.6875 |  |  |  |  | 87.32083333 | 44.6625 |
|  |  |  |  |  |  | 85.97083333 | 44.72083333 |  |  |  |  | 87.32916667 | 44.42916667 |
|  |  |  |  |  |  | 85.97083333 | 44.72916667 |  |  |  |  | 87.32916667 | 44.52916667 |
|  |  |  |  |  |  | 85.9875 | 46.4875 |  |  |  |  | 87.32916667 | 44.59583333 |
|  |  |  |  |  |  | 86.04583333 | 46.19583333 |  |  |  |  | 87.32916667 | 44.72916667 |
|  |  |  |  |  |  | 86.0875 | 41.10416667 |  |  |  |  | 87.32916667 | 47.0125 |
|  |  |  |  |  |  | 86.12083333 | 46.19583333 |  |  |  |  | 87.3375 | 44.72083333 |
|  |  |  |  |  |  | 86.12916667 | 38.52916667 |  |  |  |  | 87.3375 | 46.3625 |
|  |  |  |  |  |  | 86.17083333 | 47.82916667 |  |  |  |  | 87.3375 | 46.90416667 |
|  |  |  |  |  |  | 86.1875 | 38.55416667 |  |  |  |  | 87.34583333 | 44.44583333 |
|  |  |  |  |  |  | 86.19583333 | 46.2375 |  |  |  |  | 87.34583333 | 44.4625 |
|  |  |  |  |  |  | 86.19583333 | 46.3625 |  |  |  |  | 87.34583333 | 46.92916667 |
|  |  |  |  |  |  | 86.2125 | 41.54583333 |  |  |  |  | 87.35416667 | 46.77083333 |
|  |  |  |  |  |  | 86.2125 | 47.8625 |  |  |  |  | 87.3875 | 44.52916667 |
|  |  |  |  |  |  | 86.22083333 | 40.95416667 |  |  |  |  | 87.3875 | 44.6625 |
|  |  |  |  |  |  | 86.2875 | 46.34583333 |  |  |  |  | 87.3875 | 46.39583333 |
|  |  |  |  |  |  | 86.29583333 | 38.57083333 |  |  |  |  | 87.4125 | 44.67083333 |
|  |  |  |  |  |  | 86.32916667 | 41.10416667 |  |  |  |  | 87.4375 | 45.27083333 |
|  |  |  |  |  |  | 86.34583333 | 44.64583333 |  |  |  |  | 87.4875 | 44.6375 |
|  |  |  |  |  |  | 86.35416667 | 44.57916667 |  |  |  |  | 87.50416667 | 44.6375 |
|  |  |  |  |  |  | 86.42083333 | 41.2875 |  |  |  |  | 87.5125 | 44.62916667 |
|  |  |  |  |  |  | 86.47083333 | 41.4375 |  |  |  |  | 87.52916667 | 45.87916667 |
|  |  |  |  |  |  | 86.49583333 | 41.10416667 |  |  |  |  | 87.52916667 | 45.89583333 |
|  |  |  |  |  |  | 86.52083333 | 41.4125 |  |  |  |  | 87.52916667 | 46.52083333 |
|  |  |  |  |  |  | 86.5375 | 42.30416667 |  |  |  |  | 87.54583333 | 38.5625 |
|  |  |  |  |  |  | 86.5375 | 44.8625 |  |  |  |  | 87.54583333 | 44.6375 |
|  |  |  |  |  |  | 86.57916667 | 47.95416667 |  |  |  |  | 87.54583333 | 44.67083333 |
|  |  |  |  |  |  | 86.59583333 | 37.67916667 |  |  |  |  | 87.54583333 | 45.82916667 |
|  |  |  |  |  |  | 86.60416667 | 42.30416667 |  |  |  |  | 87.55416667 | 44.65416667 |
|  |  |  |  |  |  | 86.6125 | 38.70416667 |  |  |  |  | 87.55416667 | 45.85416667 |
|  |  |  |  |  |  | 86.67916667 | 37.69583333 |  |  |  |  | 87.57916667 | 45.7875 |
|  |  |  |  |  |  | 86.75416667 | 47.7875 |  |  |  |  | 87.57916667 | 46.1125 |
|  |  |  |  |  |  | 86.8125 | 38.70416667 |  |  |  |  | 87.5875 | 45.82083333 |
|  |  |  |  |  |  | 86.82083333 | 47.47916667 |  |  |  |  | 87.5875 | 46.15416667 |
|  |  |  |  |  |  | 86.8625 | 38.69583333 |  |  |  |  | 87.59583333 | 45.77083333 |
|  |  |  |  |  |  | 86.90416667 | 44.47083333 |  |  |  |  | 87.59583333 | 46.0375 |
|  |  |  |  |  |  | 86.92083333 | 44.47916667 |  |  |  |  | 87.60416667 | 45.97916667 |
|  |  |  |  |  |  | 86.9375 | 38.6875 |  |  |  |  | 87.6125 | 45.2375 |
|  |  |  |  |  |  | 86.94583333 | 44.57083333 |  |  |  |  | 87.6125 | 45.32083333 |
|  |  |  |  |  |  | 86.94583333 | 44.7375 |  |  |  |  | 87.62083333 | 44.5625 |
|  |  |  |  |  |  | 86.94583333 | 47.6375 |  |  |  |  | 87.62083333 | 45.27083333 |
|  |  |  |  |  |  | 86.97083333 | 38.6875 |  |  |  |  | 87.62083333 | 47.07916667 |
|  |  |  |  |  |  | 86.97916667 | 44.5625 |  |  |  |  | 87.62916667 | 44.5375 |
|  |  |  |  |  |  | 86.9875 | 43.7375 |  |  |  |  | 87.62916667 | 45.95416667 |
|  |  |  |  |  |  | 87.02083333 | 44.80416667 |  |  |  |  | 87.6375 | 44.47083333 |
|  |  |  |  |  |  | 87.02083333 | 44.82083333 |  |  |  |  | 87.64583333 | 45.42916667 |
|  |  |  |  |  |  | 87.02916667 | 44.77916667 |  |  |  |  | 87.67083333 | 44.4125 |
|  |  |  |  |  |  | 87.0375 | 44.7375 |  |  |  |  | 87.67083333 | 44.42083333 |
|  |  |  |  |  |  | 87.0375 | 44.75416667 |  |  |  |  | 87.67083333 | 45.47916667 |
|  |  |  |  |  |  | 87.05416667 | 38.70416667 |  |  |  |  | 87.67083333 | 46.1875 |
|  |  |  |  |  |  | 87.05416667 | 44.7375 |  |  |  |  | 87.67916667 | 44.42916667 |
|  |  |  |  |  |  | 87.0625 | 38.70416667 |  |  |  |  | 87.6875 | 45.72083333 |
|  |  |  |  |  |  | 87.0625 | 43.72916667 |  |  |  |  | 87.70416667 | 45.94583333 |
|  |  |  |  |  |  | 87.07916667 | 47.0375 |  |  |  |  | 87.7125 | 45.5375 |
|  |  |  |  |  |  | 87.2375 | 44.3375 |  |  |  |  | 87.72083333 | 46.20416667 |
|  |  |  |  |  |  | 87.27083333 | 38.67916667 |  |  |  |  | 87.72916667 | 46.8625 |
|  |  |  |  |  |  | 87.29583333 | 38.67916667 |  |  |  |  | 87.7375 | 46.2125 |
|  |  |  |  |  |  | 87.29583333 | 47.00416667 |  |  |  |  | 87.74583333 | 45.5875 |
|  |  |  |  |  |  | 87.3125 | 43.5375 |  |  |  |  | 87.74583333 | 46.50416667 |
|  |  |  |  |  |  | 87.32083333 | 44.6625 |  |  |  |  | 87.77916667 | 46.2375 |
|  |  |  |  |  |  | 87.32916667 | 44.65416667 |  |  |  |  | 87.7875 | 45.69583333 |
|  |  |  |  |  |  | 87.39583333 | 37.80416667 |  |  |  |  | 87.79583333 | 45.69583333 |
|  |  |  |  |  |  | 87.4125 | 44.67083333 |  |  |  |  | 87.79583333 | 47.75416667 |
|  |  |  |  |  |  | 87.54583333 | 38.5625 |  |  |  |  | 87.80416667 | 45.8375 |
|  |  |  |  |  |  | 87.57916667 | 45.94583333 |  |  |  |  | 87.80416667 | 46.27083333 |
|  |  |  |  |  |  | 87.57916667 | 46.1125 |  |  |  |  | 87.80416667 | 46.2875 |
|  |  |  |  |  |  | 87.59583333 | 44.30416667 |  |  |  |  | 87.8125 | 45.70416667 |
|  |  |  |  |  |  | 87.59583333 | 46.0375 |  |  |  |  | 87.82083333 | 45.9375 |
|  |  |  |  |  |  | 87.62083333 | 44.22916667 |  |  |  |  | 87.82916667 | 46.27083333 |
|  |  |  |  |  |  | 87.62916667 | 46.17916667 |  |  |  |  | 87.85416667 | 46.27083333 |
|  |  |  |  |  |  | 87.6875 | 44.42916667 |  |  |  |  | 87.92916667 | 45.6375 |
|  |  |  |  |  |  | 87.6875 | 45.72083333 |  |  |  |  | 87.92916667 | 45.87083333 |
|  |  |  |  |  |  | 87.7625 | 45.62083333 |  |  |  |  | 87.9375 | 44.74583333 |
|  |  |  |  |  |  | 87.77916667 | 44.3875 |  |  |  |  | 87.9375 | 45.92083333 |
|  |  |  |  |  |  | 87.7875 | 43.7625 |  |  |  |  | 87.9375 | 46.27083333 |
|  |  |  |  |  |  | 87.7875 | 45.69583333 |  |  |  |  | 87.9375 | 46.39583333 |
|  |  |  |  |  |  | 87.8125 | 45.70416667 |  |  |  |  | 87.9375 | 46.47916667 |
|  |  |  |  |  |  | 87.82083333 | 44.3625 |  |  |  |  | 87.94583333 | 46.39583333 |
|  |  |  |  |  |  | 87.85416667 | 46.27083333 |  |  |  |  | 87.95416667 | 46.35416667 |
|  |  |  |  |  |  | 87.8625 | 46.50416667 |  |  |  |  | 87.9625 | 44.37083333 |
|  |  |  |  |  |  | 87.8625 | 46.62916667 |  |  |  |  | 87.97083333 | 44.7125 |
|  |  |  |  |  |  | 87.89583333 | 44.40416667 |  |  |  |  | 87.97083333 | 46.32916667 |
|  |  |  |  |  |  | 87.90416667 | 40.45416667 |  |  |  |  | 87.97083333 | 46.64583333 |
|  |  |  |  |  |  | 87.92083333 | 38.9125 |  |  |  |  | 87.97916667 | 46.27916667 |
|  |  |  |  |  |  | 87.92916667 | 38.9125 |  |  |  |  | 87.97916667 | 46.3125 |
|  |  |  |  |  |  | 87.94583333 | 46.3625 |  |  |  |  | 87.9875 | 44.07083333 |
|  |  |  |  |  |  | 87.97083333 | 44.10416667 |  |  |  |  | 88.00416667 | 44.6875 |
|  |  |  |  |  |  | 88.0125 | 44.3875 |  |  |  |  | 88.02083333 | 44.39583333 |
|  |  |  |  |  |  | 88.0375 | 44.12916667 |  |  |  |  | 88.02916667 | 44.6625 |
|  |  |  |  |  |  | 88.0375 | 44.4625 |  |  |  |  | 88.0375 | 44.4625 |
|  |  |  |  |  |  | 88.17083333 | 39.1375 |  |  |  |  | 88.0375 | 45.77916667 |
|  |  |  |  |  |  | 88.17083333 | 44.3875 |  |  |  |  | 88.04583333 | 44.4375 |
|  |  |  |  |  |  | 88.1875 | 38.8125 |  |  |  |  | 88.04583333 | 45.6125 |
|  |  |  |  |  |  | 88.22083333 | 39.0125 |  |  |  |  | 88.05416667 | 44.62916667 |
|  |  |  |  |  |  | 88.22083333 | 44.40416667 |  |  |  |  | 88.0625 | 45.7125 |
|  |  |  |  |  |  | 88.25416667 | 40.25416667 |  |  |  |  | 88.07916667 | 45.8625 |
|  |  |  |  |  |  | 88.2625 | 44.4125 |  |  |  |  | 88.0875 | 45.90416667 |
|  |  |  |  |  |  | 88.2625 | 46.55416667 |  |  |  |  | 88.1125 | 44.62083333 |
|  |  |  |  |  |  | 88.30416667 | 39.0125 |  |  |  |  | 88.14583333 | 44.60416667 |
|  |  |  |  |  |  | 88.30416667 | 44.47916667 |  |  |  |  | 88.20416667 | 45.6375 |
|  |  |  |  |  |  | 88.30416667 | 44.4875 |  |  |  |  | 88.2375 | 44.5625 |
|  |  |  |  |  |  | 88.30416667 | 44.49583333 |  |  |  |  | 88.25416667 | 44.54583333 |
|  |  |  |  |  |  | 88.3875 | 39.54583333 |  |  |  |  | 88.2625 | 44.64583333 |
|  |  |  |  |  |  | 88.39583333 | 39.5625 |  |  |  |  | 88.27083333 | 44.5875 |
|  |  |  |  |  |  | 88.40416667 | 39.57916667 |  |  |  |  | 88.27083333 | 44.6375 |
|  |  |  |  |  |  | 88.40416667 | 39.5875 |  |  |  |  | 88.27083333 | 44.6625 |
|  |  |  |  |  |  | 88.4625 | 47.25416667 |  |  |  |  | 88.27083333 | 44.70416667 |
|  |  |  |  |  |  | 88.67083333 | 39.0375 |  |  |  |  | 88.27083333 | 44.75416667 |
|  |  |  |  |  |  | 88.7125 | 43.32083333 |  |  |  |  | 88.27916667 | 44.7875 |
|  |  |  |  |  |  | 88.72916667 | 42.34583333 |  |  |  |  | 88.29583333 | 44.82083333 |
|  |  |  |  |  |  | 88.72916667 | 43.29583333 |  |  |  |  | 88.30416667 | 44.47916667 |
|  |  |  |  |  |  | 88.7625 | 44.17083333 |  |  |  |  | 88.30416667 | 44.4875 |
|  |  |  |  |  |  | 88.82083333 | 44.1875 |  |  |  |  | 88.30416667 | 44.49583333 |
|  |  |  |  |  |  | 88.82916667 | 44.49583333 |  |  |  |  | 88.30416667 | 46.52916667 |
|  |  |  |  |  |  | 88.82916667 | 44.5625 |  |  |  |  | 88.3125 | 43.34583333 |
|  |  |  |  |  |  | 88.8375 | 44.62083333 |  |  |  |  | 88.32083333 | 44.85416667 |
|  |  |  |  |  |  | 88.84583333 | 42.72083333 |  |  |  |  | 88.32916667 | 44.52083333 |
|  |  |  |  |  |  | 88.84583333 | 42.85416667 |  |  |  |  | 88.35416667 | 44.8875 |
|  |  |  |  |  |  | 88.84583333 | 42.8625 |  |  |  |  | 88.37083333 | 44.9375 |
|  |  |  |  |  |  | 88.8625 | 39.19583333 |  |  |  |  | 88.3875 | 44.4875 |
|  |  |  |  |  |  | 88.90416667 | 44.60416667 |  |  |  |  | 88.40416667 | 44.42083333 |
|  |  |  |  |  |  | 88.92916667 | 44.62916667 |  |  |  |  | 88.4125 | 44.45416667 |
|  |  |  |  |  |  | 88.95416667 | 42.87083333 |  |  |  |  | 88.4125 | 44.9625 |
|  |  |  |  |  |  | 88.95416667 | 44.6625 |  |  |  |  | 88.4375 | 44.95416667 |
|  |  |  |  |  |  | 88.97916667 | 43.04583333 |  |  |  |  | 88.47083333 | 44.94583333 |
|  |  |  |  |  |  | 88.9875 | 44.30416667 |  |  |  |  | 88.52916667 | 47.2125 |
|  |  |  |  |  |  | 89.0125 | 43.0125 |  |  |  |  | 88.57083333 | 44.95416667 |
|  |  |  |  |  |  | 89.02916667 | 42.8125 |  |  |  |  | 88.6125 | 44.94583333 |
|  |  |  |  |  |  | 89.0375 | 42.85416667 |  |  |  |  | 88.67916667 | 44.94583333 |
|  |  |  |  |  |  | 89.05416667 | 42.7125 |  |  |  |  | 88.72083333 | 46.77916667 |
|  |  |  |  |  |  | 89.0875 | 43.9875 |  |  |  |  | 88.7375 | 46.80416667 |
|  |  |  |  |  |  | 89.09583333 | 42.90416667 |  |  |  |  | 88.7875 | 44.3875 |
|  |  |  |  |  |  | 89.12916667 | 44.6125 |  |  |  |  | 88.82916667 | 44.4875 |
|  |  |  |  |  |  | 89.15416667 | 44.47083333 |  |  |  |  | 88.82916667 | 44.5625 |
|  |  |  |  |  |  | 89.1625 | 44.42916667 |  |  |  |  | 88.82916667 | 44.57083333 |
|  |  |  |  |  |  | 89.17083333 | 44.34583333 |  |  |  |  | 88.8375 | 44.59583333 |
|  |  |  |  |  |  | 89.1875 | 42.95416667 |  |  |  |  | 88.8375 | 44.62083333 |
|  |  |  |  |  |  | 89.19583333 | 49.40416667 |  |  |  |  | 88.8375 | 46.85416667 |
|  |  |  |  |  |  | 89.25416667 | 42.69583333 |  |  |  |  | 88.84583333 | 44.67916667 |
|  |  |  |  |  |  | 89.27083333 | 42.74583333 |  |  |  |  | 88.85416667 | 44.62083333 |
|  |  |  |  |  |  | 89.29583333 | 42.8375 |  |  |  |  | 88.85416667 | 44.6875 |
|  |  |  |  |  |  | 89.3125 | 42.72916667 |  |  |  |  | 88.8625 | 44.5625 |
|  |  |  |  |  |  | 89.3375 | 45.14583333 |  |  |  |  | 88.89583333 | 44.25416667 |
|  |  |  |  |  |  | 89.35416667 | 44.0125 |  |  |  |  | 88.92916667 | 44.17083333 |
|  |  |  |  |  |  | 89.3625 | 44.04583333 |  |  |  |  | 88.99583333 | 46.95416667 |
|  |  |  |  |  |  | 89.40416667 | 42.97083333 |  |  |  |  | 89.02083333 | 45.2375 |
|  |  |  |  |  |  | 89.42916667 | 42.9375 |  |  |  |  | 89.0375 | 42.77916667 |
|  |  |  |  |  |  | 89.44583333 | 42.79583333 |  |  |  |  | 89.0375 | 42.8125 |
|  |  |  |  |  |  | 89.44583333 | 44.45416667 |  |  |  |  | 89.09583333 | 44.2125 |
|  |  |  |  |  |  | 89.4625 | 44.70416667 |  |  |  |  | 89.1375 | 44.59583333 |
|  |  |  |  |  |  | 89.4875 | 44.42916667 |  |  |  |  | 89.15416667 | 44.5125 |
|  |  |  |  |  |  | 89.52916667 | 44.67916667 |  |  |  |  | 89.15416667 | 45.00416667 |
|  |  |  |  |  |  | 89.5375 | 44.39583333 |  |  |  |  | 89.1625 | 44.45416667 |
|  |  |  |  |  |  | 89.55416667 | 38.12083333 |  |  |  |  | 89.17916667 | 44.07916667 |
|  |  |  |  |  |  | 89.60416667 | 44.62916667 |  |  |  |  | 89.19583333 | 44.7625 |
|  |  |  |  |  |  | 89.62083333 | 44.57083333 |  |  |  |  | 89.2125 | 45.05416667 |
|  |  |  |  |  |  | 89.6375 | 44.5125 |  |  |  |  | 89.2625 | 42.9125 |
|  |  |  |  |  |  | 89.64583333 | 44.4375 |  |  |  |  | 89.29583333 | 45.1125 |
|  |  |  |  |  |  | 89.6625 | 44.3125 |  |  |  |  | 89.3375 | 45.14583333 |
|  |  |  |  |  |  | 89.69583333 | 38.12083333 |  |  |  |  | 89.37083333 | 44.49583333 |
|  |  |  |  |  |  | 89.74583333 | 42.6125 |  |  |  |  | 89.37916667 | 42.7125 |
|  |  |  |  |  |  | 89.75416667 | 39.17083333 |  |  |  |  | 89.39583333 | 45.20416667 |
|  |  |  |  |  |  | 89.77083333 | 42.5375 |  |  |  |  | 89.40416667 | 44.47916667 |
|  |  |  |  |  |  | 89.79583333 | 42.57083333 |  |  |  |  | 89.4125 | 42.69583333 |
|  |  |  |  |  |  | 89.8125 | 38.15416667 |  |  |  |  | 89.42083333 | 45.2125 |
|  |  |  |  |  |  | 89.8375 | 42.7375 |  |  |  |  | 89.44583333 | 44.45416667 |
|  |  |  |  |  |  | 89.84583333 | 42.75416667 |  |  |  |  | 89.4875 | 44.42916667 |
|  |  |  |  |  |  | 89.8625 | 42.60416667 |  |  |  |  | 89.50416667 | 45.52916667 |
|  |  |  |  |  |  | 89.87083333 | 38.17083333 |  |  |  |  | 89.52916667 | 45.57083333 |
|  |  |  |  |  |  | 89.87083333 | 42.5875 |  |  |  |  | 89.5875 | 44.3625 |
|  |  |  |  |  |  | 89.87916667 | 38.17083333 |  |  |  |  | 89.5875 | 44.6875 |
|  |  |  |  |  |  | 89.97083333 | 38.20416667 |  |  |  |  | 89.6375 | 44.47916667 |
|  |  |  |  |  |  | 89.97083333 | 44.62916667 |  |  |  |  | 89.64583333 | 44.4125 |
|  |  |  |  |  |  | 89.97916667 | 44.67916667 |  |  |  |  | 89.6625 | 42.65416667 |
|  |  |  |  |  |  | 90.05416667 | 38.07083333 |  |  |  |  | 89.92916667 | 44.72916667 |
|  |  |  |  |  |  | 90.0625 | 44.2125 |  |  |  |  | 89.97083333 | 44.62916667 |
|  |  |  |  |  |  | 90.0625 | 44.39583333 |  |  |  |  | 89.97083333 | 44.6375 |
|  |  |  |  |  |  | 90.07916667 | 38.37916667 |  |  |  |  | 89.9875 | 44.57916667 |
|  |  |  |  |  |  | 90.09583333 | 44.25416667 |  |  |  |  | 90.02083333 | 44.3875 |
|  |  |  |  |  |  | 90.10416667 | 44.25416667 |  |  |  |  | 90.02083333 | 44.54583333 |
|  |  |  |  |  |  | 90.1375 | 44.1875 |  |  |  |  | 90.07083333 | 42.82083333 |
|  |  |  |  |  |  | 90.2125 | 39.02916667 |  |  |  |  | 90.07083333 | 44.22916667 |
|  |  |  |  |  |  | 90.37083333 | 38.1625 |  |  |  |  | 90.09583333 | 44.47083333 |
|  |  |  |  |  |  | 90.37916667 | 38.17916667 |  |  |  |  | 90.10416667 | 44.4125 |
|  |  |  |  |  |  | 90.39583333 | 49.50416667 |  |  |  |  | 90.1375 | 43.94583333 |
|  |  |  |  |  |  | 90.4625 | 44.14583333 |  |  |  |  | 90.17083333 | 42.8375 |
|  |  |  |  |  |  | 90.4875 | 44.15416667 |  |  |  |  | 90.19583333 | 43.9875 |
|  |  |  |  |  |  | 90.5125 | 44.20416667 |  |  |  |  | 90.20416667 | 45.64583333 |
|  |  |  |  |  |  | 90.55416667 | 44.20416667 |  |  |  |  | 90.2125 | 45.5125 |
|  |  |  |  |  |  | 90.55416667 | 44.24583333 |  |  |  |  | 90.2125 | 45.70416667 |
|  |  |  |  |  |  | 90.57916667 | 44.2625 |  |  |  |  | 90.22083333 | 45.44583333 |
|  |  |  |  |  |  | 90.6125 | 43.72916667 |  |  |  |  | 90.22916667 | 46.02083333 |
|  |  |  |  |  |  | 90.74583333 | 44.20416667 |  |  |  |  | 90.25416667 | 45.4375 |
|  |  |  |  |  |  | 90.74583333 | 44.40416667 |  |  |  |  | 90.32916667 | 44.24583333 |
|  |  |  |  |  |  | 90.7625 | 37.72083333 |  |  |  |  | 90.3625 | 46.00416667 |
|  |  |  |  |  |  | 90.77083333 | 43.24583333 |  |  |  |  | 90.42083333 | 44.04583333 |
|  |  |  |  |  |  | 90.82083333 | 44.2125 |  |  |  |  | 90.47916667 | 45.0875 |
|  |  |  |  |  |  | 90.87916667 | 44.3875 |  |  |  |  | 90.49583333 | 45.89583333 |
|  |  |  |  |  |  | 90.90416667 | 44.89583333 |  |  |  |  | 90.50416667 | 44.1625 |
|  |  |  |  |  |  | 90.94583333 | 44.4125 |  |  |  |  | 90.52083333 | 44.17083333 |
|  |  |  |  |  |  | 90.95416667 | 43.47916667 |  |  |  |  | 90.5375 | 44.19583333 |
|  |  |  |  |  |  | 90.99583333 | 44.2875 |  |  |  |  | 90.54583333 | 45.0875 |
|  |  |  |  |  |  | 90.99583333 | 45.30416667 |  |  |  |  | 90.54583333 | 45.57083333 |
|  |  |  |  |  |  | 91.02083333 | 44.42916667 |  |  |  |  | 90.54583333 | 45.80416667 |
|  |  |  |  |  |  | 91.02916667 | 44.92083333 |  |  |  |  | 90.55416667 | 44.24583333 |
|  |  |  |  |  |  | 91.07083333 | 44.45416667 |  |  |  |  | 90.57083333 | 44.25416667 |
|  |  |  |  |  |  | 91.0875 | 44.32916667 |  |  |  |  | 90.57083333 | 45.04583333 |
|  |  |  |  |  |  | 91.09583333 | 45.70416667 |  |  |  |  | 90.5875 | 45.57916667 |
|  |  |  |  |  |  | 91.09583333 | 46.10416667 |  |  |  |  | 90.59583333 | 44.2875 |
|  |  |  |  |  |  | 91.99583333 | 47.90416667 |  |  |  |  | 90.6125 | 44.3125 |
|  |  |  |  |  |  | 92.09583333 | 49.10416667 |  |  |  |  | 90.6125 | 46.17916667 |
|  |  |  |  |  |  | 92.19583333 | 45.50416667 |  |  |  |  | 90.62083333 | 44.3125 |
|  |  |  |  |  |  | 92.29583333 | 47.50416667 |  |  |  |  | 90.62083333 | 44.35416667 |
|  |  |  |  |  |  | 92.29583333 | 47.80416667 |  |  |  |  | 90.62083333 | 44.37916667 |
|  |  |  |  |  |  | 92.50416667 | 37.00416667 |  |  |  |  | 90.62083333 | 44.97083333 |
|  |  |  |  |  |  | 92.57083333 | 43.57083333 |  |  |  |  | 90.62083333 | 45.82916667 |
|  |  |  |  |  |  | 92.69583333 | 47.50416667 |  |  |  |  | 90.62916667 | 45.0125 |
|  |  |  |  |  |  | 92.9625 | 42.9375 |  |  |  |  | 90.6375 | 44.37916667 |
|  |  |  |  |  |  | 93.0625 | 42.87083333 |  |  |  |  | 90.64583333 | 44.39583333 |
|  |  |  |  |  |  | 93.10416667 | 42.8375 |  |  |  |  | 90.64583333 | 45.85416667 |
|  |  |  |  |  |  | 93.12916667 | 42.8125 |  |  |  |  | 90.6625 | 44.9875 |
|  |  |  |  |  |  | 93.17083333 | 42.77916667 |  |  |  |  | 90.69583333 | 45.67916667 |
|  |  |  |  |  |  | 93.19583333 | 37.00416667 |  |  |  |  | 90.69583333 | 45.87916667 |
|  |  |  |  |  |  | 93.22083333 | 42.75416667 |  |  |  |  | 90.70416667 | 44.40416667 |
|  |  |  |  |  |  | 93.22083333 | 43.2125 |  |  |  |  | 90.7125 | 43.4875 |
|  |  |  |  |  |  | 93.29583333 | 45.40416667 |  |  |  |  | 90.7125 | 44.37083333 |
|  |  |  |  |  |  | 93.32083333 | 42.7125 |  |  |  |  | 90.74583333 | 44.40416667 |
|  |  |  |  |  |  | 93.32083333 | 43.67916667 |  |  |  |  | 90.74583333 | 45.77916667 |
|  |  |  |  |  |  | 93.32916667 | 42.57083333 |  |  |  |  | 90.7875 | 44.3875 |
|  |  |  |  |  |  | 93.49583333 | 45.50416667 |  |  |  |  | 90.79583333 | 44.97083333 |
|  |  |  |  |  |  | 93.54583333 | 42.9875 |  |  |  |  | 90.80416667 | 43.4375 |
|  |  |  |  |  |  | 93.55416667 | 42.97083333 |  |  |  |  | 90.8125 | 45.84583333 |
|  |  |  |  |  |  | 93.57083333 | 43.02083333 |  |  |  |  | 90.8125 | 46.17916667 |
|  |  |  |  |  |  | 93.57916667 | 43.05416667 |  |  |  |  | 90.82083333 | 43.42083333 |
|  |  |  |  |  |  | 93.57916667 | 43.52083333 |  |  |  |  | 90.82916667 | 44.4125 |
|  |  |  |  |  |  | 93.59583333 | 45.30416667 |  |  |  |  | 90.85416667 | 43.3875 |
|  |  |  |  |  |  | 93.59583333 | 45.50416667 |  |  |  |  | 90.85416667 | 44.92916667 |
|  |  |  |  |  |  | 93.65416667 | 42.97083333 |  |  |  |  | 90.8625 | 43.3875 |
|  |  |  |  |  |  | 93.67083333 | 43.02916667 |  |  |  |  | 90.87916667 | 44.3875 |
|  |  |  |  |  |  | 93.69583333 | 43.07916667 |  |  |  |  | 90.87916667 | 45.9125 |
|  |  |  |  |  |  | 93.69583333 | 45.50416667 |  |  |  |  | 90.9125 | 46.07916667 |
|  |  |  |  |  |  | 93.70416667 | 43.1125 |  |  |  |  | 90.9375 | 45.95416667 |
|  |  |  |  |  |  | 93.79583333 | 45.50416667 |  |  |  |  | 90.99583333 | 44.2875 |
|  |  |  |  |  |  | 93.8125 | 42.6125 |  |  |  |  | 91.0375 | 44.3125 |
|  |  |  |  |  |  | 93.84583333 | 36.70416667 |  |  |  |  | 91.07083333 | 44.45416667 |
|  |  |  |  |  |  | 93.87083333 | 41.7375 |  |  |  |  | 91.0875 | 44.32916667 |
|  |  |  |  |  |  | 93.9625 | 42.5625 |  |  |  |  | 91.12083333 | 44.8875 |
|  |  |  |  |  |  | 94.05416667 | 42.54583333 |  |  |  |  | 91.1875 | 44.8875 |
|  |  |  |  |  |  | 94.07083333 | 42.64583333 |  |  |  |  | 91.19583333 | 44.39583333 |
|  |  |  |  |  |  | 94.14583333 | 42.6625 |  |  |  |  | 91.22083333 | 44.34583333 |
|  |  |  |  |  |  | 94.17916667 | 42.59583333 |  |  |  |  | 91.24583333 | 44.9125 |
|  |  |  |  |  |  | 94.27083333 | 42.64583333 |  |  |  |  | 91.2625 | 44.4125 |
|  |  |  |  |  |  | 94.4625 | 42.62083333 |  |  |  |  | 91.62916667 | 43.65416667 |
|  |  |  |  |  |  | 94.49583333 | 46.10416667 |  |  |  |  | 91.6875 | 43.77916667 |
|  |  |  |  |  |  | 94.5625 | 42.49583333 |  |  |  |  | 91.74583333 | 43.70416667 |
|  |  |  |  |  |  | 94.69583333 | 42.57916667 |  |  |  |  | 91.7875 | 43.7125 |
|  |  |  |  |  |  | 94.8375 | 42.52916667 |  |  |  |  | 91.89583333 | 43.77083333 |
|  |  |  |  |  |  | 94.8875 | 41.9375 |  |  |  |  | 92.17916667 | 43.87083333 |
|  |  |  |  |  |  | 94.9625 | 42.52916667 |  |  |  |  | 92.29583333 | 45.60416667 |
|  |  |  |  |  |  | 94.9875 | 42.45416667 |  |  |  |  | 92.29583333 | 45.80416667 |
|  |  |  |  |  |  | 95.0125 | 41.9875 |  |  |  |  | 92.59583333 | 44.0375 |
|  |  |  |  |  |  | 95.09583333 | 46.20416667 |  |  |  |  | 92.7375 | 43.72916667 |
|  |  |  |  |  |  | 95.15416667 | 42.0125 |  |  |  |  | 92.80416667 | 43.75416667 |
|  |  |  |  |  |  | 95.20416667 | 43.2125 |  |  |  |  | 93.0875 | 43.05416667 |
|  |  |  |  |  |  | 95.24583333 | 42.02083333 |  |  |  |  | 93.0875 | 43.07916667 |
|  |  |  |  |  |  | 95.29583333 | 42.02083333 |  |  |  |  | 93.0875 | 43.17916667 |
|  |  |  |  |  |  | 95.29583333 | 43.14583333 |  |  |  |  | 93.09583333 | 43.1125 |
|  |  |  |  |  |  | 95.29583333 | 46.30416667 |  |  |  |  | 93.1125 | 43.9125 |
|  |  |  |  |  |  | 95.3125 | 43.10416667 |  |  |  |  | 93.1375 | 43.27916667 |
|  |  |  |  |  |  | 95.3375 | 42.34583333 |  |  |  |  | 93.1375 | 43.9125 |
|  |  |  |  |  |  | 95.37916667 | 42.0125 |  |  |  |  | 93.19583333 | 45.40416667 |
|  |  |  |  |  |  | 95.3875 | 43.1875 |  |  |  |  | 93.27916667 | 43.02083333 |
|  |  |  |  |  |  | 95.4125 | 42.3125 |  |  |  |  | 93.44583333 | 43.0375 |
|  |  |  |  |  |  | 95.42083333 | 42.37916667 |  |  |  |  | 93.45416667 | 43.09583333 |
|  |  |  |  |  |  | 95.44583333 | 43.15416667 |  |  |  |  | 93.45416667 | 43.10416667 |
|  |  |  |  |  |  | 95.4625 | 42.02916667 |  |  |  |  | 93.4625 | 43.12083333 |
|  |  |  |  |  |  | 95.47083333 | 42.27083333 |  |  |  |  | 93.60416667 | 43.1125 |
|  |  |  |  |  |  | 95.52083333 | 43.19583333 |  |  |  |  | 93.6625 | 42.97083333 |
|  |  |  |  |  |  | 95.5375 | 42.05416667 |  |  |  |  | 93.74583333 | 43.15416667 |
|  |  |  |  |  |  | 95.5375 | 42.25416667 |  |  |  |  | 93.7875 | 43.12916667 |
|  |  |  |  |  |  | 95.5625 | 42.24583333 |  |  |  |  | 93.79583333 | 45.00416667 |
|  |  |  |  |  |  | 95.6875 | 42.49583333 |  |  |  |  | 94.12083333 | 43.4875 |
|  |  |  |  |  |  | 95.70416667 | 42.19583333 |  |  |  |  | 94.14583333 | 42.5875 |
|  |  |  |  |  |  | 95.77083333 | 42.14583333 |  |  |  |  | 94.15416667 | 43.37916667 |
|  |  |  |  |  |  | 96.29583333 | 44.90416667 |  |  |  |  | 94.1625 | 43.4375 |
|  |  |  |  |  |  | 96.59583333 | 44.90416667 |  |  |  |  | 94.17916667 | 42.6375 |
|  |  |  |  |  |  | 96.69583333 | 47.70416667 |  |  |  |  | 94.1875 | 42.70416667 |
|  |  |  |  |  |  | 96.70416667 | 40.12916667 |  |  |  |  | 94.1875 | 43.45416667 |
|  |  |  |  |  |  | 97.19583333 | 46.00416667 |  |  |  |  | 94.19583333 | 42.60416667 |
|  |  |  |  |  |  | 97.39583333 | 45.70416667 |  |  |  |  | 94.19583333 | 43.9625 |
|  |  |  |  |  |  | 97.62083333 | 36.65416667 |  |  |  |  | 94.22083333 | 43.4125 |
|  |  |  |  |  |  | 98.09583333 | 46.00416667 |  |  |  |  | 94.22083333 | 43.47916667 |
|  |  |  |  |  |  | 98.34583333 | 37.19583333 |  |  |  |  | 94.22916667 | 42.6375 |
|  |  |  |  |  |  | 98.99583333 | 43.20416667 |  |  |  |  | 94.22916667 | 42.72916667 |
|  |  |  |  |  |  | 98.99583333 | 43.30416667 |  |  |  |  | 94.22916667 | 43.95416667 |
|  |  |  |  |  |  | 99.19583333 | 45.50416667 |  |  |  |  | 94.24583333 | 42.74583333 |
|  |  |  |  |  |  | 99.19583333 | 45.60416667 |  |  |  |  | 94.27083333 | 42.64583333 |
|  |  |  |  |  |  | 99.29583333 | 46.10416667 |  |  |  |  | 94.27916667 | 42.77083333 |
|  |  |  |  |  |  | 99.47083333 | 39.60416667 |  |  |  |  | 94.2875 | 43.3875 |
|  |  |  |  |  |  |  |  |  |  |  |  | 94.32083333 | 42.65416667 |
|  |  |  |  |  |  |  |  |  |  |  |  | 94.3375 | 43.3875 |
|  |  |  |  |  |  |  |  |  |  |  |  | 94.34583333 | 42.67083333 |
|  |  |  |  |  |  |  |  |  |  |  |  | 94.3875 | 42.6625 |
|  |  |  |  |  |  |  |  |  |  |  |  | 94.40416667 | 43.4125 |
|  |  |  |  |  |  |  |  |  |  |  |  | 94.42916667 | 43.42916667 |
|  |  |  |  |  |  |  |  |  |  |  |  | 94.4625 | 43.4375 |
|  |  |  |  |  |  |  |  |  |  |  |  | 94.52916667 | 43.45416667 |
|  |  |  |  |  |  |  |  |  |  |  |  | 94.62916667 | 43.47083333 |
|  |  |  |  |  |  |  |  |  |  |  |  | 94.6625 | 43.32083333 |
|  |  |  |  |  |  |  |  |  |  |  |  | 94.67916667 | 43.27916667 |
|  |  |  |  |  |  |  |  |  |  |  |  | 94.67916667 | 43.29583333 |
|  |  |  |  |  |  |  |  |  |  |  |  | 94.69583333 | 43.17916667 |
|  |  |  |  |  |  |  |  |  |  |  |  | 94.7125 | 43.37916667 |
|  |  |  |  |  |  |  |  |  |  |  |  | 94.7375 | 43.14583333 |
|  |  |  |  |  |  |  |  |  |  |  |  | 94.75416667 | 43.15416667 |
|  |  |  |  |  |  |  |  |  |  |  |  | 94.75416667 | 43.42083333 |
|  |  |  |  |  |  |  |  |  |  |  |  | 94.77083333 | 43.25416667 |
|  |  |  |  |  |  |  |  |  |  |  |  | 94.77916667 | 43.17083333 |
|  |  |  |  |  |  |  |  |  |  |  |  | 94.77916667 | 43.17916667 |
|  |  |  |  |  |  |  |  |  |  |  |  | 94.77916667 | 43.20416667 |
|  |  |  |  |  |  |  |  |  |  |  |  | 94.79583333 | 43.17083333 |
|  |  |  |  |  |  |  |  |  |  |  |  | 94.82083333 | 43.1625 |
|  |  |  |  |  |  |  |  |  |  |  |  | 94.8375 | 43.1375 |
|  |  |  |  |  |  |  |  |  |  |  |  | 94.87083333 | 43.2625 |
|  |  |  |  |  |  |  |  |  |  |  |  | 94.87916667 | 43.12083333 |
|  |  |  |  |  |  |  |  |  |  |  |  | 94.90416667 | 43.15416667 |
|  |  |  |  |  |  |  |  |  |  |  |  | 94.9125 | 42.52916667 |
|  |  |  |  |  |  |  |  |  |  |  |  | 94.92916667 | 43.42916667 |
|  |  |  |  |  |  |  |  |  |  |  |  | 94.95416667 | 43.10416667 |
|  |  |  |  |  |  |  |  |  |  |  |  | 94.95416667 | 43.42916667 |
|  |  |  |  |  |  |  |  |  |  |  |  | 94.95416667 | 43.67916667 |
|  |  |  |  |  |  |  |  |  |  |  |  | 94.97916667 | 43.4125 |
|  |  |  |  |  |  |  |  |  |  |  |  | 95.00416667 | 43.0875 |
|  |  |  |  |  |  |  |  |  |  |  |  | 95.07916667 | 41.99583333 |
|  |  |  |  |  |  |  |  |  |  |  |  | 95.50416667 | 42.40416667 |
|  |  |  |  |  |  |  |  |  |  |  |  | 95.6125 | 42.07916667 |
|  |  |  |  |  |  |  |  |  |  |  |  | 95.6375 | 42.47916667 |
|  |  |  |  |  |  |  |  |  |  |  |  | 95.6625 | 42.0875 |
|  |  |  |  |  |  |  |  |  |  |  |  | 95.7125 | 42.09583333 |
|  |  |  |  |  |  |  |  |  |  |  |  | 95.84583333 | 42.52083333 |
|  |  |  |  |  |  |  |  |  |  |  |  | 96.1875 | 42.02083333 |
|  |  |  |  |  |  |  |  |  |  |  |  | 96.79583333 | 44.60416667 |
|  |  |  |  |  |  |  |  |  |  |  |  | 97.04583333 | 40.29583333 |
|  |  |  |  |  |  |  |  |  |  |  |  | 97.10416667 | 41.8625 |
|  |  |  |  |  |  |  |  |  |  |  |  | 97.19583333 | 45.80416667 |
|  |  |  |  |  |  |  |  |  |  |  |  | 98.15416667 | 39.74583333 |
|  |  |  |  |  |  |  |  |  |  |  |  | 99.50416667 | 39.00416667 |

**Table S2 Species occurrence points used in JSDM modeling**

| Przewalski’s Jird(*Brachiones przewalskii*) | Cheng’s Gerbil(*Meriones chengi*) | Libyan Jird(*Meriones libycus*) | Mid-day Gerbil(*Meriones meridianus*) | Tamarisk Gerbil(*Meriones tamariscinus*) | Mongolian Gerbil(*Meriones unguiculatus*) | Great Gerbil(*Rhombomys opimus*) | longitude | latitude |
| --- | --- | --- | --- | --- | --- | --- | --- | --- |
| 0 | 0 | 0 | 1 | 0 | 0 | 1 | 88.30416667 | 44.47916667 |
| 0 | 0 | 0 | 1 | 0 | 0 | 1 | 88.30416667 | 44.4875 |
| 0 | 0 | 0 | 1 | 0 | 0 | 1 | 88.30416667 | 44.49583333 |
| 0 | 0 | 0 | 1 | 0 | 1 | 0 | 78.99583333 | 43.00416667 |
| 0 | 0 | 0 | 1 | 0 | 1 | 0 | 78.99583333 | 44.00416667 |
| 0 | 0 | 0 | 1 | 0 | 0 | 1 | 85.97083333 | 44.72083333 |
| 0 | 0 | 0 | 1 | 0 | 0 | 1 | 85.97083333 | 44.72916667 |
| 0 | 0 | 1 | 1 | 0 | 0 | 0 | 87.0375 | 44.7375 |
| 0 | 0 | 1 | 0 | 0 | 0 | 1 | 87.0375 | 44.74583333 |
| 0 | 0 | 1 | 1 | 0 | 0 | 1 | 87.0375 | 44.75416667 |
| 0 | 0 | 1 | 0 | 0 | 1 | 1 | 74.99583333 | 44.00416667 |
| 0 | 0 | 1 | 0 | 0 | 1 | 0 | 74.99583333 | 45.00416667 |
| 0 | 0 | 1 | 0 | 0 | 1 | 0 | 76.99583333 | 45.00416667 |
| 0 | 0 | 1 | 1 | 0 | 0 | 1 | 82.29583333 | 45.00416667 |
| 0 | 0 | 1 | 1 | 0 | 0 | 1 | 82.29583333 | 45.10416667 |
| 1 | 0 | 0 | 1 | 0 | 0 | 1 | 87.54583333 | 38.5625 |
| 0 | 0 | 1 | 1 | 0 | 0 | 0 | 88.84583333 | 42.8625 |
| 0 | 0 | 0 | 1 | 0 | 1 | 0 | 92.29583333 | 47.50416667 |
| 0 | 0 | 0 | 1 | 0 | 1 | 0 | 92.29583333 | 47.80416667 |
| 0 | 0 | 0 | 1 | 0 | 1 | 1 | 101.9958333 | 43.90416667 |
| 0 | 0 | 0 | 1 | 0 | 1 | 0 | 101.9958333 | 44.70416667 |
| 1 | 0 | 0 | 1 | 0 | 0 | 0 | 85.49583333 | 38.30416667 |
| 0 | 0 | 0 | 1 | 0 | 0 | 1 | 111.0958333 | 42.50416667 |
| 0 | 0 | 1 | 1 | 0 | 0 | 0 | 86.5375 | 44.8625 |
| 0 | 0 | 1 | 0 | 0 | 0 | 1 | 87.59583333 | 45.77083333 |
| 0 | 0 | 0 | 1 | 0 | 0 | 1 | 87.59583333 | 46.0375 |
| 0 | 0 | 0 | 1 | 0 | 0 | 1 | 88.82916667 | 44.5625 |
| 0 | 0 | 0 | 1 | 1 | 0 | 0 | 91.09583333 | 45.70416667 |
| 0 | 0 | 0 | 1 | 1 | 0 | 0 | 91.09583333 | 46.10416667 |
| 1 | 0 | 0 | 1 | 0 | 0 | 0 | 80.3625 | 37.27083333 |
| 0 | 0 | 1 | 0 | 0 | 1 | 0 | 73.00416667 | 43.00416667 |
| 0 | 0 | 1 | 0 | 0 | 1 | 0 | 73.00416667 | 44.00416667 |
| 0 | 0 | 1 | 0 | 0 | 0 | 1 | 82.42083333 | 45.5625 |
| 1 | 0 | 0 | 1 | 0 | 0 | 1 | 82.8125 | 37.34583333 |
| 0 | 0 | 1 | 1 | 0 | 0 | 1 | 83.04583333 | 44.62083333 |
| 0 | 0 | 0 | 1 | 0 | 0 | 1 | 85.37916667 | 46.05416667 |
| 0 | 0 | 0 | 1 | 0 | 0 | 1 | 85.37916667 | 46.0625 |
| 0 | 0 | 0 | 1 | 1 | 0 | 0 | 87.2375 | 44.3375 |
| 0 | 0 | 0 | 1 | 0 | 0 | 1 | 88.0375 | 44.4625 |
| 0 | 0 | 0 | 1 | 0 | 0 | 1 | 88.8375 | 44.62083333 |
| 0 | 1 | 1 | 1 | 0 | 0 | 0 | 89.09583333 | 42.90416667 |
| 0 | 0 | 1 | 1 | 0 | 0 | 1 | 90.55416667 | 44.24583333 |
| 0 | 0 | 0 | 1 | 0 | 0 | 1 | 90.74583333 | 44.40416667 |
| 0 | 0 | 0 | 1 | 0 | 1 | 0 | 93.59583333 | 45.30416667 |
| 0 | 0 | 0 | 1 | 0 | 1 | 0 | 93.59583333 | 45.50416667 |
| 0 | 0 | 0 | 1 | 0 | 1 | 0 | 93.69583333 | 45.50416667 |
| 0 | 0 | 0 | 1 | 0 | 1 | 0 | 103.9958333 | 43.50416667 |
| 0 | 0 | 0 | 1 | 0 | 1 | 0 | 104.0958333 | 43.50416667 |
| 1 | 0 | 1 | 1 | 0 | 0 | 0 | 77.1125 | 39.5625 |
| 0 | 0 | 1 | 1 | 0 | 1 | 0 | 77.99583333 | 46.00416667 |
| 1 | 0 | 0 | 1 | 0 | 0 | 0 | 79.99583333 | 37.30416667 |
| 0 | 0 | 1 | 1 | 0 | 0 | 1 | 82.49583333 | 45.00416667 |
| 0 | 0 | 0 | 1 | 0 | 0 | 1 | 86.34583333 | 44.64583333 |
| 0 | 0 | 1 | 1 | 1 | 0 | 0 | 86.35416667 | 44.57916667 |
| 0 | 0 | 0 | 1 | 0 | 0 | 1 | 86.94583333 | 44.57083333 |
| 0 | 0 | 1 | 1 | 0 | 0 | 1 | 87.4125 | 44.67083333 |
| 0 | 0 | 0 | 1 | 0 | 0 | 1 | 87.57916667 | 46.1125 |
| 0 | 0 | 0 | 1 | 0 | 0 | 1 | 87.6875 | 45.72083333 |
| 0 | 0 | 1 | 1 | 0 | 0 | 1 | 87.7875 | 45.69583333 |
| 0 | 0 | 0 | 1 | 1 | 0 | 1 | 87.8125 | 45.70416667 |
| 0 | 0 | 1 | 1 | 0 | 0 | 0 | 88.95416667 | 42.87083333 |
| 0 | 0 | 1 | 0 | 0 | 0 | 1 | 89.0375 | 42.8125 |
| 0 | 0 | 1 | 1 | 0 | 0 | 1 | 89.3375 | 45.14583333 |
| 0 | 0 | 1 | 0 | 0 | 0 | 1 | 90.57083333 | 44.25416667 |
| 0 | 0 | 0 | 1 | 0 | 0 | 1 | 90.99583333 | 44.2875 |
| 0 | 0 | 0 | 1 | 0 | 1 | 1 | 101.4958333 | 45.20416667 |
| 0 | 0 | 0 | 1 | 0 | 1 | 1 | 102.2958333 | 43.80416667 |
| 0 | 0 | 0 | 1 | 0 | 1 | 0 | 103.6958333 | 47.40416667 |
| 0 | 0 | 1 | 0 | 0 | 1 | 0 | 104.9958333 | 37.50416667 |
| 0 | 0 | 0 | 1 | 0 | 1 | 0 | 106.7958333 | 44.80416667 |
| 1 | 0 | 0 | 1 | 0 | 0 | 0 | 77.47083333 | 37.65416667 |
| 1 | 0 | 0 | 1 | 0 | 0 | 0 | 77.7875 | 38.6625 |
| 1 | 0 | 0 | 1 | 0 | 0 | 0 | 79.60416667 | 37.5625 |
| 0 | 1 | 0 | 1 | 0 | 0 | 0 | 80.39583333 | 44.20416667 |
| 0 | 0 | 0 | 1 | 1 | 0 | 0 | 82.19583333 | 46.40416667 |
| 0 | 0 | 1 | 0 | 0 | 0 | 1 | 82.40416667 | 45.5625 |
| 0 | 0 | 0 | 1 | 0 | 0 | 1 | 82.82916667 | 44.45416667 |
| 1 | 0 | 0 | 1 | 0 | 0 | 0 | 82.8875 | 37.62916667 |
| 0 | 0 | 0 | 1 | 0 | 0 | 1 | 82.97083333 | 45.02083333 |
| 0 | 0 | 0 | 1 | 1 | 0 | 0 | 85.09583333 | 47.90416667 |
| 0 | 0 | 0 | 1 | 0 | 0 | 1 | 85.34583333 | 46.02083333 |
| 0 | 0 | 1 | 0 | 0 | 0 | 1 | 86.02916667 | 44.0125 |
| 0 | 0 | 1 | 0 | 0 | 0 | 1 | 86.10416667 | 46.8375 |
| 0 | 0 | 1 | 0 | 0 | 0 | 1 | 86.17083333 | 46.7375 |
| 0 | 0 | 1 | 0 | 0 | 0 | 1 | 86.2625 | 46.7875 |
| 0 | 0 | 1 | 1 | 0 | 0 | 0 | 87.02083333 | 44.80416667 |
| 0 | 0 | 0 | 1 | 0 | 0 | 1 | 87.32083333 | 44.6625 |
| 0 | 0 | 1 | 0 | 0 | 0 | 1 | 87.74583333 | 46.50416667 |
| 0 | 0 | 0 | 1 | 0 | 0 | 1 | 87.85416667 | 46.27083333 |
| 0 | 0 | 1 | 0 | 0 | 0 | 1 | 87.97916667 | 46.27916667 |
| 0 | 0 | 1 | 1 | 0 | 0 | 0 | 89.02916667 | 42.8125 |
| 0 | 0 | 0 | 1 | 0 | 0 | 1 | 89.4875 | 44.42916667 |
| 0 | 0 | 0 | 1 | 0 | 0 | 1 | 89.44583333 | 44.45416667 |
| 0 | 0 | 0 | 1 | 0 | 0 | 1 | 89.6375 | 44.47916667 |
| 0 | 0 | 1 | 1 | 0 | 0 | 0 | 89.8125 | 38.15416667 |
| 0 | 0 | 1 | 1 | 0 | 0 | 0 | 89.8625 | 42.60416667 |
| 0 | 0 | 0 | 1 | 0 | 0 | 1 | 89.97083333 | 44.62916667 |
| 0 | 0 | 1 | 0 | 0 | 0 | 1 | 90.20416667 | 45.64583333 |
| 0 | 0 | 0 | 1 | 0 | 0 | 1 | 90.87916667 | 44.3875 |
| 0 | 0 | 0 | 1 | 0 | 0 | 1 | 91.07083333 | 44.45416667 |
| 0 | 0 | 0 | 1 | 0 | 0 | 1 | 91.0875 | 44.32916667 |
| 0 | 0 | 0 | 0 | 1 | 0 | 1 | 91.19583333 | 44.39583333 |
| 0 | 0 | 1 | 0 | 0 | 0 | 1 | 91.2625 | 44.4125 |
| 0 | 0 | 0 | 1 | 1 | 0 | 0 | 92.19583333 | 45.50416667 |
| 0 | 0 | 0 | 1 | 0 | 0 | 1 | 94.27083333 | 42.64583333 |
| 0 | 0 | 0 | 1 | 0 | 1 | 0 | 94.49583333 | 46.10416667 |
| 0 | 0 | 0 | 1 | 0 | 1 | 0 | 95.09583333 | 46.20416667 |
| 0 | 0 | 0 | 1 | 0 | 1 | 0 | 95.29583333 | 46.30416667 |
| 0 | 0 | 0 | 1 | 0 | 1 | 0 | 99.29583333 | 46.10416667 |
| 0 | 0 | 0 | 1 | 0 | 1 | 0 | 103.7958333 | 43.60416667 |
| 0 | 0 | 0 | 1 | 0 | 1 | 0 | 104.2958333 | 47.80416667 |
| 0 | 0 | 0 | 1 | 0 | 1 | 0 | 107.2958333 | 45.80416667 |
| 0 | 1 | 0 | 0 | 0 | 0 | 0 | 88.77916667 | 43.0875 |
| 0 | 1 | 0 | 0 | 0 | 0 | 0 | 87.8375 | 43.52083333 |
| 0 | 1 | 0 | 0 | 0 | 0 | 0 | 88.07083333 | 43.5625 |
| 0 | 1 | 0 | 0 | 0 | 0 | 0 | 75.94583333 | 39.85416667 |
| 0 | 1 | 0 | 0 | 0 | 0 | 0 | 76.1875 | 40.10416667 |
| 0 | 1 | 0 | 0 | 0 | 0 | 0 | 76.0375 | 40.1125 |
| 0 | 1 | 0 | 0 | 0 | 0 | 0 | 76.74583333 | 40.15416667 |
| 0 | 1 | 0 | 0 | 0 | 0 | 0 | 55.10416667 | 45.90416667 |
