# Supplementary material for: Environmental Limitations and Interspecific Interactions Across the Distribution Range of Gerbils
Source: Ecol Evol. 2025 Nov 10;15(11):e72468. doi: 10.1002/ece3.72468 (PMC12602260; doi:10.1002/ece3.72468)
Supplement: Supplementary file 2 — Data S1: ece372468‐sup‐0002‐Supinfo.docx. [file ECE3-15-e72468-s001.docx]

**Supplementary Materials**

**Table S3 Environmental variables associated with species distribution**

| **Factor** | **Spatialresolution** | **Sources** |
| --- | --- | --- |
| (bio-1)Annual mean temperature | 30s | WorldClim |
| (bio-2)Mean diurnal range | 30s | WorldClim |
| (bio-3)Isothermality | 30s | WorldClim |
| (bio-4)Temperature seasonality | 30s | WorldClim |
| (bio-5)Max temperature of warmest month | 30s | WorldClim |
| (bio-6)Min Temperature of coldest month | 30s | WorldClim |
| (bio-7)Temperature annual range | 30s | WorldClim |
| (bio-8)Mean temperature of wettest quarter | 30s | WorldClim |
| (bio-9)Mean temperature of driest quarter | 30s | WorldClim |
| (bio-10)Mean temperature of warmest quarter | 30s | WorldClim |
| (bio-11)Mean temperature of coldest quarter | 30s | WorldClim |
| (bio-12)Annual precipitation | 30s | WorldClim |
| (bio-13)Precipitation of wettest month | 30s | WorldClim |
| (bio-14)Precipitation of driest month | 30s | WorldClim |
| (bio-15)Precipitation seasonality | 30s | WorldClim |
| (bio-16)Precipitation of wettest quarter | 30s | WorldClim |
| (bio-17)Precipitation of driest quarter | 30s | WorldClim |
| (bio-18)Precipitation of warmest quarter | 30s | WorldClim |
| (bio-19)Precipitation of coldest quarter | 30s | WorldClim |
| Altitude | 30s | WorldClim |
| Slope | 30s | WorldClim |
| Aspect | 30s | WorldClim |
| Grazing intensity | 30s | Aerospace Information Research Institute, CAS |
| Nighttime light index | 30s | UEMM team |
| Human footprint index | 30s | UEMM team |

**Table S4 AUC values and TSS values of Maxent model**

| Species | Training AUC | Test AUC | Training TSS | Test TSS |
| --- | --- | --- | --- | --- |
| Przewalski’s Jird  (*Brachiones przewalskii*) | 0.998 | 0.994 | 0.752 | 0.534 |
| Cheng’s Gerbil  (*Meriones chengi*) | 0.996 | 0.919 | 0.841 | 0.960 |
| Libyan Jird  (*Meriones libycus*) | 0.989 | 0.953 | 0.521 | 0.319 |
| Mid-day Gerbil  (*Meriones meridianus*) | 0.939 | 0.921 | 0.439 | 0.428 |
| Tamarisk Gerbil  (*Meriones tamariscinus*) | 0.988 | 0.937 | 0.503 | 0.393 |
| Mongolian Gerbil  (*Meriones unguiculatus*) | 0.980 | 0.896 | 0.452 | 0.334 |
| Great Gerbil  (*Rhombomys opimus*) | 0.953 | 0.932 | 0.339 | 0.324 |

**Joint Species Distribution Model Script**

install.packages("jSDM")

library(jSDM)

#library(metafor)

data<-read.csv("I:/2Gerbillinae/zhongyaGerbillinae/20250802.csv")

head(data)

# Species presence/absence information

PA <- data[,19:25]

head(PA)

# Environmental variables

Env <- data[,1:18]

head(Env)

# Calculate the number of ecological process variables

np <- ncol(Env)

# Number of sampling sites

nsite <- 120

# Number of species

nsp<- 7

# Set latent variables

n_latent <- 2

# Set latent variables using longitude and latitude

W <- data[,26:27]

head(W)

# Species effect: refers to the degree of influence of different species on the environment or ecosystem.

beta.target <- t(matrix(runif(nsp*np,-2,2),

byrow=TRUE, nrow=nsp))

# Factor loadings: parameters in a statistical model describing the relationship between latent and observed variables.

# Factor loadings describe the extent to which latent variables affect observed variables, i.e., their associations.

lambda.target <- matrix(0, n_latent, nsp)

mat <- t(matrix(runif(nsp*n_latent, -2, 2), byrow=TRUE, nrow=nsp))

lambda.target[upper.tri(mat, diag=TRUE)] <- mat[upper.tri(mat, diag=TRUE)]

dim(Env) # 100 x 3

dim(beta.target) # 7 x 3

dim(W) # 100 x 2

dim(lambda.target) # 2 x 7

length(alpha.target) # 100

diag(lambda.target) <- runif(n_latent, 0, 2)

# Variance of random site effects

V_alpha.target <- 0.5

# Random effect

# Residual variance of fixed-effect model / residual variance of random-effect model

alpha.target <- rnorm(nsite,0 , sqrt(V_alpha.target))

# Use probit link function to simulate response data.

# The probit link function is commonly used for binary or multinomial logistic regression problems where the dependent variable is binary.

Env <- as.matrix(Env, nrow=nsite, ncol=np)

W <- as.matrix(W, nrow=nsite, ncol=n_latent)

probit_theta <- Env%*%beta.target + W%*%lambda.target + alpha.target

theta <- pnorm(probit_theta)

# Model fitting

mod<-jSDM_binomial_probit(

burnin=200,# Change number of iterations

mcmc=200,

thin=1,# Interval between iterations

presence_data=PA,# Species data

site_formula=~.,# Environmental variables

site_data = Env,# Environmental variable data

n_latent=2,

site_effect="random",

alpha_start=0,

beta_start=0,

lambda_start=0,

W_start=0,

V_alpha=1,

shape_Valpha=0.5,

rate_Valpha=0.0005,

mu_beta=0, V_beta=1,

mu_lambda=0, V_lambda=1,

seed=1234, verbose=1)

# Parameter estimation

oldpar <- par(no.readonly = TRUE)

# Plot trace and density plots of species beta_j coefficients

mean_beta <- matrix(0,nsp,ncol(Env))

pdf(file="I:/2Gerbillinae/1/beta_j_paginated.pdf", width=10, height=15)

plots_per_page <- 6

plot_idx <- 0

for (j in 1:nsp) {

for (p in 1:ncol(Env)) {

if (plot_idx %% plots_per_page == 0) {

par(mfrow=c(3,2), mar=c(4,4,2,1))

}

plot_idx <- plot_idx + 1

coda::traceplot(mod$mcmc.sp[[j]][, p], main=paste(colnames(mod$mcmc.sp[[j]])[p], ", species:", j))

}

}

dev.off()

write.csv(mean_beta, file = "I:/2Gerbillinae/1/mean_beta.csv", row.names = FALSE)

## Plot trace and density plots of species lambda_j parameters

mean_lambda <- matrix(0,nsp,n_latent)

pdf(file="I:/2Gerbillinae/1/lambda_j.pdf")

par(mfrow=c(n_latent*2,2))

for (j in 1:nsp) {

mean_lambda[j,] <- apply(mod$mcmc.sp[[j]]

[,(ncol(Env)+1):(ncol(Env)+n_latent)], 2, mean)

for (l in 1:n_latent) {

coda::traceplot(mod$mcmc.sp[[j]][,ncol(Env)+l])

coda::densplot(mod$mcmc.sp[[j]][,ncol(Env)+l],

main=paste(colnames(mod$mcmc.sp[[j]])

[ncol(Env)+l],", species : ",j))

abline(v=lambda.target[l,j],col='red')

}

}

dev.off()

write.csv(mean_lambda, file = "I:/2Gerbillinae/1/mean_lambda.csv", row.names = FALSE)

# Species effects and factor loadings

par(mfrow=c(1,2))

plot(t(beta.target), mean_beta,

main="species effect beta",

xlab ="obs", ylab ="fitted")

abline(a=0,b=1,col='red')

plot(t(lambda.target), mean_lambda,

main="factor loadings lambda",

xlab ="obs", ylab ="fitted")

abline(a=0,b=1,col='red')

write.csv(summary(mod$mcmc.alpha)[[1]], file = "I:/2Gerbillinae/1/alpha_summary.csv")

## Plot trace and density plots of latent variables Wi

pdf(file="I:/2Gerbillinae/1/Wi.pdf")

par(mfrow=c(1,2))

for (l in 1:n_latent) {

plot(W[,l],

summary(mod$mcmc.latent[[paste0("lv_",l)]])[[1]][,"Mean"],

main = paste0("Latent variable W_", l),

xlab ="obs", ylab ="fitted")

abline(a=0,b=1,col='red')

}

dev.off()

## alpha, site effects

par(mfrow=c(1,3))

plot(alpha.target, summary(mod$mcmc.alpha)[[1]][,"Mean"],

xlab ="obs", ylab ="fitted", main="site effect alpha")

abline(a=0,b=1,col='red')

## Trace and density plots of V_alpha

coda::traceplot(mod$mcmc.V_alpha)

coda::densplot(mod$mcmc.V_alpha)

abline(v=V_alpha.target,col='red')

## Trace and density plots of model deviance

summary(mod$mcmc.Deviance)

plot(mod$mcmc.Deviance)

# Prediction

par(mfrow=c(1,2))

plot(probit_theta, mod$logit_theta_latent,

main="probit_theta)",

xlab="obs", ylab="fitted")

abline(a=0 ,b=1, col="red")

plot(theta, mod$theta_latent,

main="Probabilities of occurence theta",

xlab="obs", ylab="fitted")

abline(a=0 ,b=1, col="red")

par(oldpar)

# Model residuals

plot_residual_cor(mod)

# Species association plot

# Residual correlations between species

R <- get_residual_cor(mod)$cor.mean

plot_associations(R, circleBreak = TRUE, occ = PA, species_order="abundance")

# Bayesian MCMC sample means of species environmental effects (beta)

env_effect <- t(sapply(mod$mcmc.sp,

colMeans)[grep("beta_", colnames(mod$mcmc.sp[[1]]))[-1],])

colnames(env_effect) <- gsub("beta_", "", colnames(env_effect))

plot_associations(R, env_effect = env_effect, species_order="main env_effect")

# Model-based prediction

# Select sites and species for prediction

## Select 30 sites

Id_sites <- sample.int(nrow(PA), 100)

## Select five species

Id_species <- sample(colnames(PA), 7)

# Prediction

theta_pred <- predict(mod,

Id_species=Id_species,

Id_sites=Id_sites,

type="mean")

# Plot histogram

hist(theta_pred, main="Predicted theta with simulated covariates")

theta_pred

write.csv(theta_pred, file = "I:/2Gerbillinae/1/theta_pred.csv", row.names = FALSE)

# Calculate mean of residual correlation matrix

R <- get_residual_cor(mod)$cor.mean

# Add species names to rows and columns of species correlation matrix (assuming PA column names are species names)

species_names <- colnames(PA)

colnames(R) <- species_names

rownames(R) <- species_names

# Export to CSV

write.csv(R, file = "I:/2Gerbillinae/1/species_residual_correlation.csv", row.names = TRUE)

# Extract mean matrix of beta environmental effects

env_effect <- t(sapply(mod$mcmc.sp,

colMeans)[grep("beta_", colnames(mod$mcmc.sp[[1]]))[-1],])

colnames(env_effect) <- gsub("beta_", "", colnames(env_effect))

rownames(env_effect) <- colnames(PA)

write.csv(env_effect, file = "I:/2Gerbillinae/1/species_env_effect.csv", row.names = TRUE)

**Maxent Model Optimization Script**

library("reshape2") # Data reshaping package, convenient for data transformation

library("SDMtune") # Species distribution model tuning and evaluation package

library("ENMeval") # MaxEnt model evaluation package

library("plotROC") # Draw ROC curves

library("ggplot2") # Plotting package

library("dismo") # Ecological distribution modeling package

library("raster") # Raster data processing package

library("sp") # Spatial data processing package

library("sf") # Modern spatial data processing package

library("dplyr") # Data manipulation package

library("maps") # Map drawing package

library("MASS") # Statistical analysis package

library("zeallot") # Support multiple variable assignment

library("virtualspecies") # Virtual species simulation

library("rJava") # R interface to Java

# Check current working directory, confirm file location

getwd()

# Set working directory (adjust according to your folder path)

# MaxEnt program, environmental variables, and species occurrence data should be placed in this folder

setwd("I:/2Gerbillinae/zhongyaGerbillinae/0maxent")

# Load environmental raster variables (climatic variables, soil, etc.)

# Here .asc format files are used, each file represents one variable

bio1 <- raster("bio1.asc")

bio2 <- raster("bio2.asc")

...

bio19 <- raster("bio19.asc")

bio20 <- raster("biohb.asc") # Elevation

bio21 <- raster("biopd.asc") # Slope

bio22 <- raster("biopx.asc") # Aspect

bio23 <- raster("fmqd.asc") # Grazing intensity

bio24 <- raster("nl.asc") # Nighttime lights

bio25 <- raster("rlzj.asc") # Human footprint

# Combine all environmental rasters into a single stack

env <- stack(bio1, bio2, ..., bio25)

# raster::raster() and terra::rast() cannot be mixed directly; convert to terra format for processing

envtest <- terra::rast(env)

# Generate 10,000 random background points from the full environmental extent using SDMtune

bg2 <- terra::spatSample(envtest,

size = 1000,

method = "random",

na.rm = TRUE,

xy = TRUE,

values = FALSE)

# Load species occurrence points (CSV format)

# Assume first column is index; remove it and keep coordinates

occ <- read.csv("Meriones meridianus.csv")[,-1]

# Convert occurrence points to raster (cells with presence = 1)

occur.ras <- rasterize(occ, env, 1)

# Plot rasterized occurrence points to check species distribution range

plot(occur.ras)

# Extract indices of raster cells with value = 1 (presence cells)

presences <- which(values(occur.ras) == 1)

# Get coordinates of species presence points

pres.locs <- coordinates(occur.ras)[presences, ]

# Use 2D kernel density estimation to calculate presence density, for creating a bias file

dens <- kde2d(pres.locs[,1], pres.locs[,2],

n = c(nrow(occur.ras), ncol(occur.ras)),

lims = c(extent(env)[1], extent(env)[2], extent(env)[3], extent(env)[4]))

# Convert kernel density results to raster format, matching environmental extent and resolution

dens.ras <- raster(dens, env)

dens.ras2 <- resample(dens.ras, env)

# Save bias file for MaxEnt background point weighting (to reduce sampling bias)

writeRaster(dens.ras2, "biasfile.asc", overwrite = TRUE)

# Count non-NA cells in environmental raster

length(which(!is.na(values(subset(env, 1)))))

# Sample 10,000 background points weighted by bias file

bgnew <- xyFromCell(dens.ras2, sample(which(!is.na(values(subset(env, 1)))), 10000,

prob=values(dens.ras2)[!is.na(values(subset(env, 1)))]))

# Make background point column names consistent with occurrence points

colnames(bgnew) <- colnames(occ)

# Prepare species-environment dataset using prepareSWD, combining presence and background points

datatest <- prepareSWD(species = "Litylenchus species",

p = occ,

a = bgnew,

env = envtest)

# Save prepared dataset for later use

swd2csv(datatest, file_name = "datatest.csv")

# Train default MaxEnt model using SWD data

model <- train(method = "Maxent", data = datatest)

# Check model slot names

slotNames(model)

# Calculate model evaluation metrics

auc(model) # AUC

tss(model) # TSS

aicc(model, env = envtest) # AICc (requires environmental variables)

# Split data into training and testing (20% test set), split only presences, set random seed = 25

c(train, test) %<-% trainValTest(datatest,

test = 0.2,

only_presence = TRUE,

seed = 25)

# Train Maxnet model using training set (alternative MaxEnt implementation)

maxnet_model <- train("Maxnet", data = train)

# Check Maxnet model slot names

slotNames(maxnet_model)

# Print AUC for training and test sets

cat("Training auc: ", auc(maxnet_model))

cat("Testing auc: ", auc(maxnet_model, test = test))

# Plot ROC curve for test set

plotROC(maxnet_model, test = test)

# Compute TSS and AICc

tss(maxnet_model)

aicc(maxnet_model, env = envtest)

# Check slots of internal Maxnet model

slotNames(maxnet_model@model)

# Generate random k-fold cross-validation folds (k=10), only splitting presences, seed=25

folds <- randomFolds(datatest, k = 10, only_presence = TRUE, seed = 25)

# Train Maxent model with cross-validation folds

cv_model <- train("Maxent", data = datatest, folds = folds)

# Calculate variable importance

vi <- maxentVarImp(cv_model)

vi

# Plot variable importance (columns 1 and 2)

plotVarImp(vi[, 1:2])

# Plot permutation importance (columns 1 and 3)

plotVarImp(vi[, c(1,3)])

# Prepare dataset containing only background points

bgtest <- prepareSWD(species = "Litylenchus species",

a = bgnew,

env = envtest)

# Variable selection based on AUC, remove highly correlated variables (Spearman, cor_th = 0.8)

selected_variables_model <- varSel(cv_model,

metric = "auc",

test = test,

bg4cor = bgtest,

method = "spearman",

cor_th = 0.8,

permut = 1)

exists("selected_variables_model")

# Check selected model slots

slotNames(selected_variables_model@models)

# Export selected variables dataset

swd2csv(selected_variables_model@data, file_name = "selected_variables_model_data.csv")

# Select subset of environmental variables, rebuild raster stack

envselect <- stack(bio2, bio3, bio7, bio13, bio15, bio19, bio21, bio22, bio25)

# Set environment variable path (replace with actual path)

env_dir <- "I:/2Gerbillinae/zhongyaGerbillinae/0maxent"

# Load all .asc files as terra SpatRaster

env_files <- list.files(env_dir, pattern = ".asc$", full.names = TRUE)

envselect <- rast(env_files)

class(envselect)

# Expected output:

# [1] "SpatRaster"

# Use ENMevaluate to tune model parameters for selected variables

# fc: feature classes

# rm: regularization multipliers, from 0.5 to 4, step = 0.5

enmeval_results1123 <- ENMevaluate(

occs = occ,

envs = envselect,

bg = bgnew,

tune.args = list(

fc = c("L", "LQ", "H", "LQH", "LQHP", "LQHPT"),

rm = seq(0.5, 4, 0.5)

),

partitions = "randomkfold",

partition.settings = list(kfolds = 10),

algorithm = "maxnet"

)

# Predict habitat suitability using best model

best_pred_raster <- predict(

object = envselect,

model = best_model,

filename = "best_model_prediction.tif", # Output path

type = "cloglog", # Recommended Maxent output type

overwrite = TRUE,

wopt = list(

datatype = "FLT4S", # Single precision float, saves space

gdal = c("BIGTIFF=YES"), # Enable large file writing

chunksize = 1e6 # Max 1 million pixels per block (adjust to memory)

)

)

# Select best model using delta.AICc

best_model <- enmeval_results1123@models[[which.min(enmeval_results1123@results$delta.AICc)]]

best_pred <- predict(envselect, best_model, type = "cloglog")

# Save model results

saveRDS(enmeval_results1123, model_result_file)

message("✅ Model completed, results saved as RDS file!")

# Extract delta.AICc for model comparison

delta_AICc3_1123 <- evalplot.stats(e = enmeval_results1123,

stats = c("delta.AICc"),

color = "fc",

x.var = "rm",

error.bars = FALSE)

# Plot delta.AICc results

print(delta_AICc3_1123)

# View detailed tuning results table

enmeval_results1123@results

# Save tuning results as CSV for reporting

write.csv(enmeval_results1123@results, "enmeval_results_1123.csv")
